# Exploring salicylic acid biosynthesis in *Trichoderma* spp. using an enhanced transformation approach

**DOI:** 10.1186/s40694-026-00208-0

**Published:** 2026-02-10

**Authors:** Siebe Pierson, Erwann Arc, Thomas Roach, Clara Baldin, Mario Gründlinger, Maximilian Mick, Patrick Herzog, Ilse Kranner, Susanne Zeilinger

**Affiliations:** 1 Department of Microbiology, Universität Innsbruck, Technikerstraße 25d, Innsbruck, Austria; 2 Department of Botany, Universität Innsbruck, Sternwartestraße 15, Innsbruck, Austria

**Keywords:** Salicylic acid, *Trichoderma*, *Trichoderma virens*, CRISPR/Cas9, Genetic transformation, UHPLC MS/MS, Gene deletion

## Abstract

**Background:**

Salicylic acid (SA) is an important plant hormone but is also produced by microorganisms. Contrary to the well-described roles and biosynthetic pathways of SA in plants, its role in fungal physiology and its biosynthesis within fungi remains largely unclear. Here, we sought to investigate the role of SA in the physiology of *Trichoderma* spp. and to identify fungal genes responsible for SA biosynthesis in *Trichoderma virens*, while applying and optimizing a transformation approach recently adapted for *Trichoderma atroviride*.

**Results:**

Significant strain- and species-dependent differences in both SA biosynthesis and growth in the presence of exogenous SA were observed. Furthermore, in certain *Trichoderma* species SA biosynthesis turned out to be induced by the presence of plant volatile organic compounds (VOCs). Based on plant SA biosynthesis pathways, candidate fungal SA biosynthesis genes were screened and respective *T. virens* gene deletion mutants generated through application and optimization of an enhanced transformation approach. Gene deletion did not result in a decrease in SA biosynthesis, providing evidence that SA biosynthesis in *T. virens* is distinct from the canonical plant pathways.

**Conclusions:**

Although we were not able to identify genes responsible for SA biosynthesis in *T. virens*, we uncovered how certain *Trichoderma* and fungal phytopathogen species are affected by SA in their environment and how SA release by *Trichoderma* spp. can be affected by the presence of a plant host. Furthermore, we were able to optimize an approach to measuring phytohormones produced by *Trichoderma* spp. in plate culture and proved the applicability of an optimized transformation approach in *T. virens*.

**Supplementary Information:**

The online version contains supplementary material available at 10.1186/s40694-026-00208-0.

## Background

Sustainable agriculture has increasingly turned to biological solutions for enhancing plant growth and managing diseases, with species of the fungal genus *Trichoderma* being at the forefront of these efforts [1]. Several *Trichoderma* species are renowned for their beneficial interactions with plants. Their colonization of plant roots as avirulent plant symbionts enhances plant stress tolerance, seed germination, plant growth, nutrient uptake, fertilizer use efficiency, and plant immune response. In addition, the mycoparasitic lifestyle of *Trichoderma* fungi can protect plants by antagonizing fungal plant pathogens [2–7]. The success of *Trichoderma* in plant protection is largely dependent on a complex cross-talk between the fungus and its plant symbiont, a communication mediated by diverse biochemical signals with phytohormones as key players [8, 9].

Phytohormones are critical regulators of plant development, growth and defence [10]. These small signalling molecules play an essential role in modulating plant responses to environmental stimuli, including interactions with beneficial microorganisms [8, 9, 11–13]. Interestingly, several *Trichoderma* spp. also produce phytohormones, which appear to trigger signalling cascades in the plant, potentially contributing to some plant-beneficial effects [8, 11, 12, 14–16].

Among the phytohormones, salicylic acid (SA) is particularly noteworthy with regard to plant-microbe interactions due to its pivotal role in plant immune responses. SA is a key player in the systemic acquired resistance (SAR) pathway, which is crucial for plant defence against a broad spectrum of pathogens. Additionally, SA plays an essential role in plant stress tolerance through modulation of antioxidative enzyme activities [12, 17–19]. *Trichoderma virens*, along with other *Trichoderma* spp. such as *T. harzianum*, *T. longibrachiatum*, *T. parareesei*, *T. spirale*, and *T. simmonsii*, has been shown to release SA in liquid culture. Phytohormones could be integral to both *Trichoderma*-plant communication [16, 20, 21] and *Trichoderma* physiology independent of plant interaction such as the ability to antagonize host fungi. For example, in *T. parareesei* and *T. harzianum*, SA production has been linked to improved antifungal capacity against *Fusarium oxysporum* and *Fusarium graminearum*, respectively [22, 23]. SA produced by the plant appears to be essential for limiting the degree of colonization by *Trichoderma*. Alonso-Ramírez et al. [24] found that the absence of SA synthesis in *A. thaliana sid2* mutants allowed *T. harzianum* to colonize vascular tissues and the aboveground parts of the plant, leading to plant collapse. Upon regular SA synthesis by the plant, optimal plant colonization by *Trichoderma* spp. requires downregulation of the pathways responsible for SA production within the plant [25–27]. Hence, the ability of *Trichoderma* spp. to also produce SA appears counter-intuitive and is difficult to reconcile within this process, highlighting the need for more in-depth investigation.

In plants, SA is synthesized through two main pathways: the isochorismate pathway (IC) and the phenylalanine ammonia-lyase (PAL) pathway. Although both pathways are usually involved in SA biosynthesis, their relative importance varies between plant species. In rice, the PAL pathway seems to be more important while in *A. thaliana*, the IC pathway is mainly responsible for SA biosynthesis. In soybean, both pathways contribute equally to SA production [28–30]. The IC pathway starts within plastids with the conversion of chorismate to isochorismate through the activity of isochorismate synthase (ICS) [31]. The isochorismate is then transported outside the plastids by a transporter encoded by the *enhanced disease susceptibility 5* (*EDS5*) gene [30, 32, 33]. In the cytosol, isochorismate is then converted to isochorismate-9-glutamate, catalysed in *A. thaliana* by the avrPphB SUSCEPTIBLE3 (PBS3) enzyme [30, 34, 35]. In other plant species, the gene responsible for this step has not yet been characterised. In the final step of the IC pathway, isochorismate-9-glutamate is converted to SA, which is catalysed by ENHANCED PSEUDOMONAS SUSCEPTIBILITY 1 (EPS1) but can also occur spontaneously [30, 36].

The PAL pathway starts with PAL catalysing the conversion of phenylalanine to trans-cinnamic acid [28]. Phenylalanine is produced via the shikimate pathway, where it is synthesized from chorismate by chorismate mutase (CM) [37]. The conversion of trans-cinnamic acid to benzoic acid is catalysed by abnormal inflorescence meristem 1 (AIM1) [38, 39]. In the final step of the PAL pathway, a presumed benzoic acid hydroxylase is believed to catalyse the conversion of benzoic acid to salicylic acid, but the role of this enzyme in SA biosynthesis has not yet been clearly characterized [28]. Contrary to the well-characterized pathways of SA biosynthesis in plants, the corresponding mechanisms in *Trichoderma* are much less understood, creating a compelling need to investigate whether the biosynthetic pathways are conserved.

Here, we sought to investigate the genetic basis of SA biosynthesis in *T. virens* by targeting orthologues of plant genes involved in SA production. We achieved this by employing and optimizing a CRISPR/Cas9-based approach for gene knockout (KO), an improvement to the traditional PEG-mediated protoplast transformation [40]. Traditionally, for *Trichoderma* spp., transformation of foreign DNA is achieved through generation of protoplasts which import DNA molecules upon treatment with polyethylene glycol (PEG). These DNA molecules often contain a gene conferring resistance to hygromycin B for subsequent transformant selection, flanked by DNA sequences homologous to the target locus that allow homologous recombination (HR). The exchange of the gene of interest (GOI) with the hygromycin B resistance-encoding marker gene (*hph-*cassette) results in gene KO and enables selection of gene deletion mutants [41, 42]. However, in a more recent and refined approach, initially developed for *Botrytis cinerea* [43] and later optimized for *T. atroviride* [40], this replacement step is bypassed. Instead, the GOI is directly excised through the action of a Cas9 endonuclease, which introduces double-strand breaks at carefully selected recognition sites flanking the GOI [44]. Hygromycin B resistance of the transformants is provided by the transiently stable pTEL vector which contains the *hph*-cassette flanked by telomeres that allow autonomous replication within the fungal cells. The pTEL vector behaves as a centromere-free minichromosome within the cell but only remains stable in the presence of selection pressure. Cultivating the mutants in the absence of hygromycin B will therefore lead to the loss of pTEL and hence the *hph*-cassette, thereby permitting recycling of the selection marker and allowing deletion of multiple genes with the same selection marker system [43, 45].

In this study, we aimed to enhance our understanding of the role of SA in the physiology of *T. virens* while applying and further optimizing a new genetic transformation approach. First, we assessed the effect of exogenous SA on *Trichoderma* and potential host fungi. Second, we quantified SA released by *Trichoderma* spp. into solid medium. Third, we applied and optimized a CRISPR/Cas9-based gene deletion approach in *T. virens* that was then employed to explore the genetic basis of SA biosynthesis. Candidate genes were identified through a BLAST search using plant genes known to participate in SA biosynthesis. Overall, we highlight the necessity to investigate the role of phytohormones in the physiology of *Trichoderma* spp. and provide an improved method for the generation of fungal gene deletion mutants.

## Materials and methods

### Strains and culture conditions

Four *Trichoderma* strains and two strains of fungal phytopathogens were used in this study: *Trichoderma atroviride* P1, *Trichoderma atroviride* IMI 206040, *Trichoderma asperellum* CBS 433.97, *Trichoderma virens* Gv29-8, *Fusarium oxysporum* f. sp. *lycopersici*, and *Rhizoctonia solani*. *A. thaliana* Col-0 was used as a plant partner. Fungi were cultivated on potato dextrose agar (PDA, BD Difco, Franklin Lakes, NJ, USA) or ‘PNM9’ medium, prepared by adding 0.5% glucose to modified PNM minimal medium, previously optimized by Johnson et al. [46] to allow for optimal growth of *A. thaliana* and *Serendipita indica* in solid culture. All fungal cultivations were carried out at 25 °C under a 12 h/12 h light/dark cycle (20 µmol m^− 2^ s^− 1^). *A. thaliana* cultivation was carried out at 22 °C under a 16 h/8 h light/dark cycle (80–100 µmol m^− 2^ s^− 1^). Co-cultivation of *A. thaliana* and *Trichoderma* was carried out at 25 °C under a 12 h/12 h light/dark cycle at low daytime light intensity (25 µmol m^− 2^ s^− 1^) to omit potential stress induced in *Trichoderma* due to light exposure.

### Salicylic acid growth assay

**Fig. 1:**
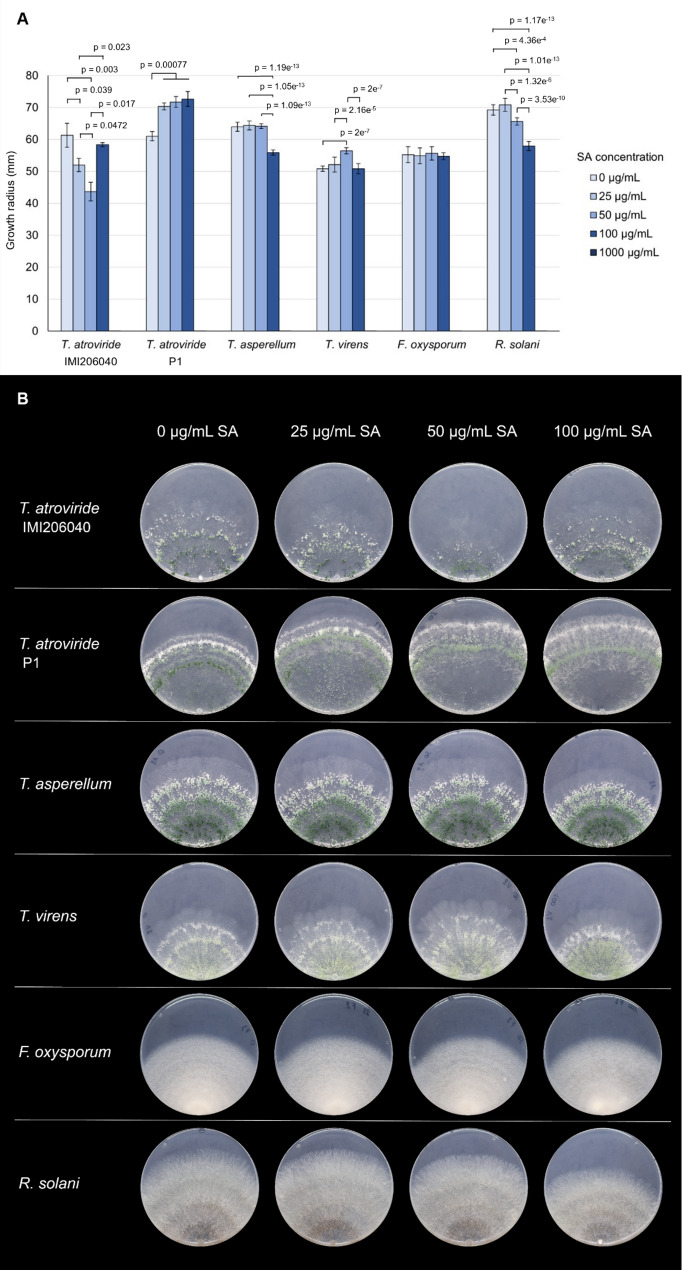
SA growth assay. Growth of four *Trichoderma* strains (*T. atroviride* IMI 206040, *T. atroviride* P1, *T. asperellum* and *T. virens*) and two phytopathogenic fungi (*F. oxysporum* and *R. solani*) on PNM9 medium containing 25–1000 µg mL^-1^ SA or ethanol as solvent control (0 µg/mL SA). **A** The bar plot shows the average colony radius (n = three replicates) achieved after six days of cultivation for the *T. atroviride* strains, five days for *T. asperellum*, *T. virens* and *R. solani*, and ten days for *F. oxysporum*. Error bars represent the standard deviation. Statistically significant differences between treatments within the same strain are indicated with brackets and p-values. **B** A representative colony for each condition is shown below the bar plot to assess colony morphology (excluding 1000 µg mL^-1^)

*Trichoderma* spp. and phytopathogenic fungi were cultivated on PNM9 medium containing SA. Growth on PNM9 with 25 µg mL^− 1^ (181 µM), 50 µg mL^− 1^ (362 µM), 100 µg mL^− 1^ (724 µM) and 1000 µg mL^− 1^ (7.24 mM) SA were compared to control medium without SA, which contained only ethanol as the solvent. Fungi were pre-cultured to reach the same developmental stage and were inoculated on the edge of the petri dish using mycelial plugs. Growth radius was measured daily with a ruler along the central axis of the plate and along two additional axes, each angled 30° from the central axis. The optimal growth duration was chosen based on the growth rate of the respective fungus. The growth radius of *T. atroviride* IMI 206040 and P1 shown in Fig. 1 was achieved in 6 days. *T. asperellum*,* T. virens* and *R. solani* were cultivated for 5 days and *F. oxysporum* for 10 days. Three replicates were cultivated per fungal strain and per SA concentration and the complete assay was repeated twice.

### Culture preparation and harvest for SA quantification

SA quantification was performed twice: first, to compare four *Trichoderma* strains across three cultivation setups, and second, to compare wildtype (WT) *T. virens* with mutants missing candidate SA biosynthesis genes. The three cultivation setups consisted of (i) axenic fungal cultures, (ii) co-cultivation of both a *Trichoderma* strain and *A. thaliana* in the same petri dish achieving physical interaction (co-culture), and (iii) a dish sandwich design which allowed the fungus to interact with the volatile organic compounds (VOCs) produced by *A. thaliana* without physical contact (split-culture). This dish sandwich design was adapted from Lazazzara et al. [47] and consisted of cultures of the fungus and plant in separate petri dishes that were sealed together (supplementary Figure S1). SA production between *T. virens* WT and mutants was compared in axenic fungal cultures including two independent gene deletion mutants for each gene.

Fungi were cultured in 60 × 15 mm petri dishes containing 4 mL PNM9 medium covered with a cellophane layer for separation of the fungal biomass from the growth medium (*n* = 5 biological replicates). *Trichoderma* spp. were inoculated by placing a drop containing 5000 conidia in the middle of the petri dish. Additionally, five blank samples were prepared in the exact same way, but with the addition of sterile water instead of spores. Plates were wrapped with parafilm to prevent drying out and reduce the loss of VOCs in the split-cultures.

*A. thaliana* seeds were surface sterilized by immersion in a 50% bleach solution for 10 min, followed by six rinses with distilled water [48]. One seed was placed in the middle of each uninoculated co-culture or split-culture plate and stratified for 3 days at 4 °C. Before fungal inoculation, *A. thaliana* was pre-grown for 10 days by vertically incubating the plate at a 65° angle to allow root growth along the agar surface. After fungal inoculation, all cultures were incubated for 6 days after which the cellophane was removed and the growth medium was harvested. Fungal dry weight (DW) was measured using an analytical balance and the growth medium was lyophilized for 120 h for assessing SA concentration.

### UHPLC-MS/MS analysis

Extracellular SA was quantified through ultra-high performance liquid chromatography-tandem mass spectrometry (UHPLC‐MS/MS) as previously described by Pichler et al. [49] with minor modifications. After addition of 75 µL ultra-pure water (UPW) and sonication, extracts were centrifuged at 26,000*g* for 10 min at 4 °C to reduce the risk of debris blocking the 0.2 μm PTFE filters used in the final step of sample preparation. All chemicals used for analysis were of the highest purity (HPLC or LC-MS-grade) and obtained from Sigma-Aldrich (Vienna, Austria) or VWR Chemicals (Vienna, Austria). All equipment used for culturing, harvesting, or UHPLC-MS/MS analysis was thoroughly rinsed three times with LC-MS-grade acetonitrile or UPW, as appropriate, and subsequently dried in a fume hood before use. After filtering, the extracts were injected into the ekspert ultra LC100 UHPLC system (Eksigent, Dublin, CA, USA) coupled to a QTRAP 4500 mass spectrometer (ABSCIEX, Framingham, MA, USA) and analysed as described by Pichler et al. [49]. Small amounts of SA were detected in the five blank samples for both SA quantifications and the mean SA value was subtracted from the SA amounts found in the biological samples. This procedure was performed for the axenic *Trichoderma* cultures, the *A. thaliana* and *Trichoderma* co-cultures and the *Trichoderma* side of the split-cultures.

### BLAST and phylogenetic analysis of SA biosynthesis genes

Protein sequences of enzymes involved in SA biosynthesis were retrieved from three plant species: *A. thaliana*, rice (*Oryza sativa*), and soybean (*Glycine max*) [28, 50]. These species were selected for their differences in favouring the PAL (rice) or IC (*A. thaliana*) pathway for SA production or combining both (soybean). Sequences were used as queries in a protein-protein BLAST (pBLAST, NCBI, Bethesda, MD, USA) search to identify orthologues in *T. atroviride* IMI 206040, *T. asperellum* CBS 433.97, *T. virens* Gv29-8, and *T. harzianum* CBS 226.95 [51]. The pBLAST analysis was performed with default parameters and a threshold E-value of 1e^− 6^, ensuring that only significant hits were considered. The protein sequences for the SA biosynthesis enzymes were obtained from publicly available genome databases (www.arabidopsis.org, rice.uga.edu, www.soybase.org) and identifiers are given in supplementary Table S1.

Sequences relevant for phylogenetic analysis were identified using pBLAST (pBLAST, NCBI Bethesda, MD, USA), either within the *Trichoderma* taxon for intra-genus comparisons, or across fungal, plant and bacterial taxa to assess evolutionary conservation. Retrieved sequences were aligned using MAFFT v. 7 [52] and the resulting alignment was checked using Mega X [53]. The best Maximum Likelihood (ML) model was calculated in IQ-TREE version 3 [54, 55]. Maximum Parsimony based bootstrap analyses were applied, setting the number of replicates to 1000. The resulting phylogenetic trees were analysed and graphically improved using iTOL [56].

Phylogenetic analyses were complemented with an examination of conserved domains for the proteins of interest across species. Protein sequences were annotated using InterPro 107.0 [57] and the identified domains were combined with the multiple sequence alignment in Jalview 2.11.5.1 [58].

### Genetic transformation

Gene deletion was performed in *T. virens* for a putative PAL (Protein ID TRIVIDRAFT_67832) and EPS1 (Protein ID TRIVIDRAFT_47880) -encoding gene orthologue. Gene sequences were retrieved from JGI Mycocosm (mycocosm.jgi.doe.gov). CRISPR RNA (crRNA) design was performed as previously described by Gruendlinger et al. [40]. Briefly, crRNAs were designed to target sequences near the 5’ and 3’ regions of the GOI to induce a deletion of the target locus (sequences provided in supplementary Table S2). The IDT internal custom gRNA tool (eu.idtdna.com) and ChopChop (chopchop.cbu.uib.no) were used for crRNA design. Proposed crRNA sequences were used in a Blastn search against *T. virens* to prevent unintended off-target DNA editing. CrRNAs, tracrRNA and SpCas9 nuclease were purchased from Integrated DNA Technologies (IDT, Coralville, IA, USA).

For fungal transformation, the procedure previously described for *T. atroviride* P1 by Gruendlinger et al. [40] was followed with a slightly adapted protoplast preparation approach. Protoplasts of *T. virens* were generated by inoculating Sabouraud dextrose agar (SAB) medium with *T. virens* conidia at a final concentration of 1 × 10^6^ conidia mL^− 1^. The resulting conidial germlings were transferred to a buffering solution (0.6 M KCl, 50 mM CaCl_2_, 5 mM Tris-HCl) containing 50 mg mL^− 1^ VinoTaste^®^ Pro (Novozym, Bagsværd, Denmark) and incubated for 1.5 h at 30°C in an orbital shaking incubator (75 rpm). Next, protoplasts were filtered out and washed as described by Gruendlinger et al. [40]. Protoplasts were counted and diluted to a concentration of 5 × 10^6^ protoplasts mL^− 1^. Next, ribonucleoprotein (RNP) complexes consisting of crRNA, tracrRNA and SpCas9 were assembled. For optimization purposes, two SpCas9 quantities were tested. Instead of using 27 µg Cas9 as previously described [45], 6 µg and 1 µg Cas9 were employed for comparison, and the crRNA and tracrRNA quantities were adjusted accordingly. The final transformation mixture for each Cas9 quantity consisted of the 5’ and 3’ RNP complexes, 10x Cas9 nuclease reaction buffer (New England Biolabs, Ipswich, MA, USA), 2x STC buffer (100 mM CaCl_2_, 2 M sorbitol, 20 mM Tris-HCl pH 7.5), 5 µg pTEL plasmid and the protoplasts. After treatment with PEG, the protoplasts were plated on medium containing 200 µg mL^− 1^ hygromycin B (Calbiochem, CA, USA) to allow transformant selection. Emerging transformants were genotyped using DreamTaq DNA polymerase (Thermo Fisher Scientific, Waltham, MA, USA) and the primers provided in supplementary Table S2. Primers and plasmids were traced using MyLabStocks [59]. This protocol was performed in parallel with different Cas9 quantities to allow for comparison. The transformation for each gene and Cas9 quantity was performed twice at different timepoints to ensure reproducibility. For each GOI, two independent mutants were selected and cultivated on PDA until they lost the pTEL vector. The mutants were then purified to mitotic stability by single spore isolation on PDA. Purified mutants were once again genotyped to confirm the absence of the GOI.

### Assay proving loss of pTEL vector

The ability of the mutants to eliminate the pTEL vector was assessed through several cycles of cultivation on non-selective medium. Gene deletion mutants were grown on PDA containing 200 µg mL^− 1^ hygromycin B until sporulation. Next, spores from the edge of the plate were transferred to PDA without hygromycin B and once again incubated until sporulation (first cycle of cultivation). Spores from the edge of this plate were then transferred to both, a PDA plate with selection pressure (200 µg mL^− 1^ hygromycin B) to test whether the pTEL vector had been lost, and a PDA plate without hygromycin B to confirm viability of the inoculated spores and start the next cycle (second cycle of cultivation). This process was repeated until the transferred spores did not germinate on PDA containing hygromycin B and the number of cycles was recorded.

### Statistical analysis

To assess differences among groups, data were first tested for normality using the Shapiro-Wilk test and for homogeneity of variances using Bartlett’s test. When the data met the assumptions of normality and equal variances, a one-way ANOVA was performed. In cases where variances were unequal, Welch’s ANOVA was applied. For data that did not follow a normal distribution, the Kruskal-Wallis test was used. Post-hoc analyses were conducted where significant differences were identified. Tukey’s Honest Significant Difference (HSD) test was used following one-way ANOVA, while the Games-Howell multiple comparisons method was applied after Welch’s ANOVA. For the Kruskal-Wallis test, pairwise comparisons were performed using the Wilcoxon rank-sum test with Benjamini-Hochberg correction to account for multiple testing. A detailed description of the statistical tests used for each dataset is given in supplementary Table S3. p-values and statistical analyses were performed using R [60], and statistical significance was set at *p* < 0.05. Box plots were created in R using the package ggpubr [61] and bar plots were created in Excel (Microsoft corporation, Redmond, WA, USA).

## Results

### Effect of SA on fungal growth

To determine how salicylic acid (SA) influences fungal growth, four *Trichoderma* strains and two phytopathogenic fungi, *F. oxysporum* and *R. solani*, were tested. The presence of SA in the growth medium had diverse effects on fungal colony growth (Fig. 1). *T. atroviride* P1 increased its growth radius upon exposure to a SA concentration range of 25 µg mL^− 1^ to 100 µg mL^− 1^, while *T. virens* only did so at 50 µg mL^−^1 SA. Apart from the increased colony size, these colonies were morphologically identical to the colonies not exposed to SA (Fig. 1). Interestingly, *T. atroviride* IMI 206040 reacted differently from strain P1, with decreased growth at 25 µg mL^− 1^ and 50 µg mL^− 1^ SA but colony extension similar to the control upon exposure to 100 µg mL^− 1^ SA. *F. oxysporum* and *T. asperellum* appeared largely unaffected by SA concentrations up to 100 µg mL^− 1^, with a decrease in colony growth for *T. asperellum* at 100 µg mL^− 1^ SA only. *R. solani* showed a stepwise decrease in growth at 50 µg mL^− 1^ and 100 µg mL^− 1^ SA with a significantly smaller colony radius than the control and the lower previous concentration. A SA concentration of 1000 µg mL^− 1^ resulted in complete growth inhibition for all fungal species tested. Despite the effects on colony extension, no differences in colony morphology were observed throughout the assay (Fig. 1).

### Quantification of fungal SA production upon growth in solid culture

**Fig. 2:**
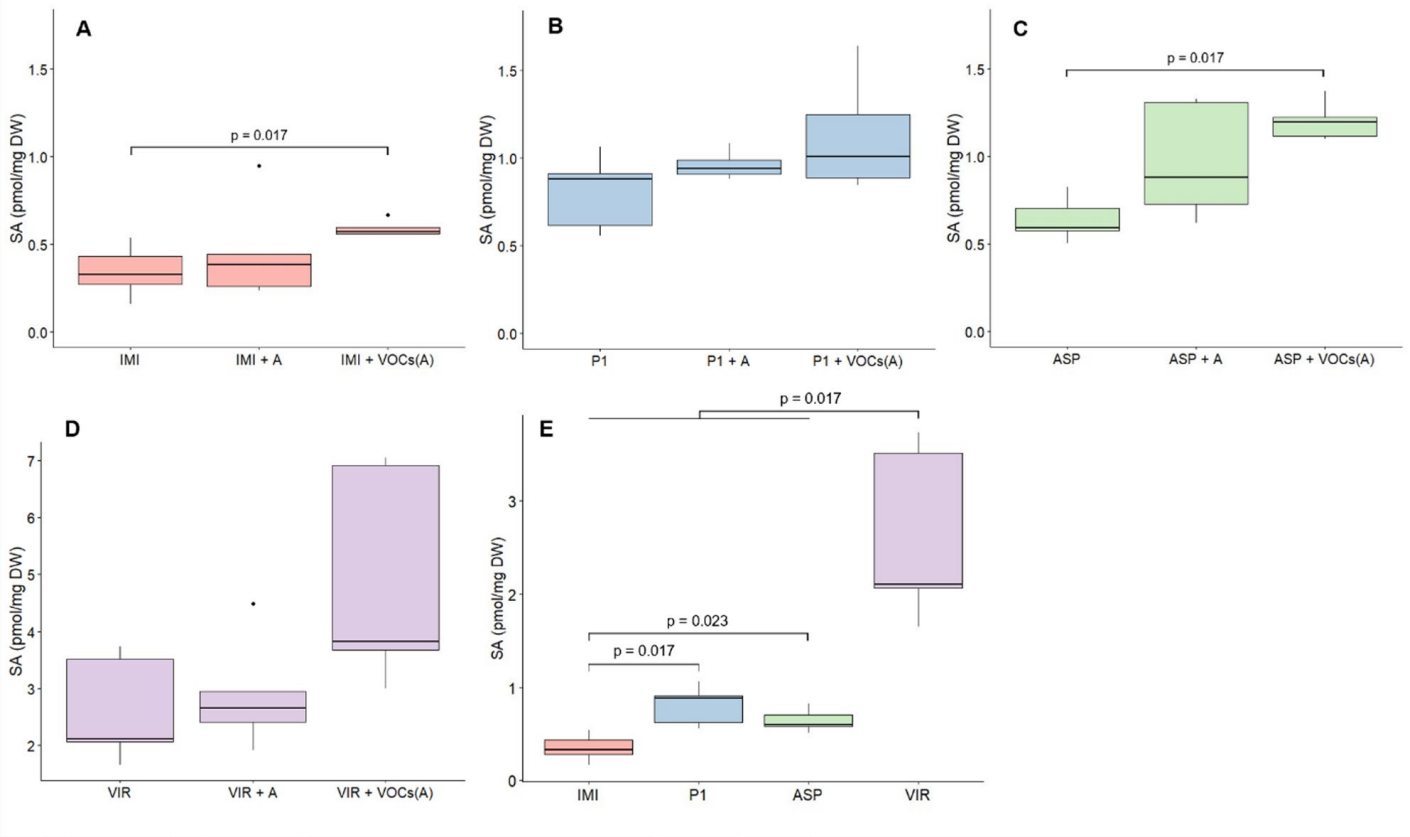
SA quantification in solid cultures. SA released into the growth medium by **A ***T. atroviride* IMI 206040 (IMI), **B ***T. atroviride* P1 (P1), **C ***T. asperellum* (ASP) and **D ***T. virens* (VIR), cultivated in axenic culture (IMI/P1/ASP/VIR), co-culture with *A. thaliana* (IMI/P1/ASP/VIR + A) or split-culture with *A. thaliana* (IMI/P1/ASP/VIR + VOCs(A)). **E** Comparison of SA levels released by the different *Trichoderma* strains in axenic culture. Statistically significant differences are indicated with brackets and p-values. Boxplots show median, 25% and 75% percentiles, maximum and minimum values, and outliers (dots) (*n* = 5)

In order to better understand how and why *Trichoderma* spp. synthesize SA, SA release in solid medium was quantified by UHPLC-MS/MS analysis in three cultivation setups. Significant differences in extracellular SA levels between *Trichoderma* strains and between cultivation setups (Fig. 2) were identified. On solid PNM9 medium, *T. virens* released about three times more SA compared to the other *Trichoderma* strains tested (Fig. 2, E). Surprisingly, the two *T. atroviride* strains, P1 and IMI 206040, released significantly different amounts of extracellular SA. In the comparison between cultivation setups (Fig. 2, A-D), an increase in SA release was observed upon volatile interaction with *A. thaliana*. For *T. atroviride* IMI 206040 and *T. asperellum*, interaction with plant VOCs led to a significant increase in extracellular SA levels (Fig. 2, A and C), while the other strains showed a clear, though non-significant, upward trend (Fig. 2, B and D).

### Search for putative genes responsible for SA production by BLAST and phylogenetic analysis

pBLAST analysis was performed in order to identify if protein orthologues of plant SA biosynthesis pathway components are present in *Trichoderma* spp. Table 1 shows a condensed summary of the output of the pBLAST search. For each plant SA biosynthesis enzyme, the best hit (lowest E-value) obtained for the different *Trichoderma* strains tested, is shown in the table with its JGI catalogue ID, coverage percentage and E-value. Interestingly, no orthologues for PAL could be found in the genomes of *T. harzianum* and *T. asperellum*, whereas the multiple paralogs of this protein within each of the three plant species all returned a single putative orthologue for *T. atroviride* and *T. virens* (see supplementary Table S1). Similarly, the same putative orthologue was identified within each *Trichoderma* species for multiple ICS paralogs. Both CM and EPS1 returned a single putative orthologue whereas the queries with AIM1 returned several hits for each *Trichoderma* spp. No orthologues were found in any of the *Trichoderma* spp. for EDS5 or PBS3.

Identified PAL and EPS1 orthologues were then used to conduct phylogenetic analyses. Initially a *Trichoderma* intra-genus investigation was carried out, using identified orthologues from *T. atroviride* and *T. virens* for a pBLAST search in the *Trichoderma* taxid. Almost identical results were obtained; therefore to avoid redundancy in the figures, only the trees obtained using *T. virens* PAL and EPS1 orthologues are shown (Figure S2, A – B). Figure S2A highlights the clustering of the *T. virens* PAL orthologue with a protein from *Trichoderma velutinum*, annotated as phenylalanine and histidine ammonia-lyase, while the orthologue from *T. atroviride* is part of a bigger cluster containing hypothetical proteins from several strains as well as L-Aspartase-like proteins. A different cluster organization was instead underlined for the EPS1 orthologues (Figure S2B). In this case, the *T. atroviride* protein clustered together with *T. asperellum* in a smaller group, while those of *T. virens* and *T. harzianum* are part of a larger cluster. However, annotation for EPS1 orthologues was not as informative, generally referring to transferase family domain-containing proteins. To gather more insight, the phylogenetic analysis was expanded across kingdoms, including retrieved sequences from fungi, bacteria and plants. The sequences of PAL and EPS1 proteins of *A. thaliana*,* O. sativa and G. max* were included (even when not identified by pBLAST search). For the PAL sequences, the three kingdoms formed distinct, equally distributed monophyletic clusters, implying a deep, early evolutionary divergence (Figure S3). For EPS1, no bacterial orthologues were identified, with the exception of a metagenome-assembled genome (MAG) (Figure S4). However, species in the fungal kingdom were not clustering closely together, indicating a broader diversity.

To complement the phylogenetic analysis, protein domain conservation was investigated across the retrieved orthologues. The same protein sequences used for phylogenetic tree reconstruction were analysed using InterPro 107.0 [57], integrating prediction from all member databases. For the PAL orthologues, the characteristic PAL-HAL lyase-aromatic domain was clearly identified in plant species. In contrast, fungal and bacterial sequences showed only partial similarity, retaining limited consensus with residues corresponding to the annotated active site (Figure S5). Notably, the orthologues from *Trichoderma viride*, *Thermomyces duportii* and both analysed strains of *T. virens* lacked conserved residues within the region associated with the active site.

In the case of EPS1 orthologues, the detected domain was broadly classified as “transferase”. InterPro identified domains included fungal, and specifically *Trichoderma* species. With the exception of a single hypothetical protein sequence from *T. harzianum*, all other orthologues retained conserved residues within the predicted active site (Figure S6). While these results are indicative of a conserved transferase-related activity, they do not provide sufficient information to infer substrate specificity or additional functional features, rendering any evolutionary divergence discussion purely speculative.

Nevertheless, the putative orthologue of PAL (TRIVIDRAFT_67832) and the putative orthologue of EPS1 (TRIVIDRAFT_47880) were selected for functional characterization in *T. virens*. The genes encoding these proteins are hereafter referred to as *virPAL* and *virEPS1*, respectively.

**Table 1:** Condensed summary of the pBLAST analysis results

*Trichoderma* species		PAL pathway		IC pathway			
		PAL	AIM1	ICS	EDS5	PBS3	EPS1
*T. harzianum*	JGI cat. ID	-	510064	112315	-	-	505728
	% Coverage		38	58			87
	E-value		1.00 × 10^− 36^	4.00 × 10^− 26^			1.00 × 10^− 11^
*T. asperellum*	JGI cat. ID	-	190397	189343	-	-	57229
	% Coverage		38	58			87
	E-value		9.00 × 10^− 35^	9.00 × 10^− 26^			1.00 × 10^− 12^
*T. atroviride*	JGI cat. ID	260476	267956	297768	-	-	146943
	% Coverage	70	38	58			88
	E-value	4.00 × 10^− 123^	3.00 × 10^− 35^	5.00 × 10^− 27^			7.00 × 10^− 11^
*T. virens*	JGI cat. ID	**67832**	211402	84460	-	-	**47880**
	% Coverage	23	38	58			87
	E-value	3.00 × 10^− 17^	1.00 × 10^− 34^	8.00 × 10^− 26^			2.00 × 10^− 12^

pBLAST analysis was performed in *T. harzianum* CBS 226.95, *T. asperellum* CBS 433.97, *T. atroviride* IMI 206040 and *T. virens* Gv29-8 with protein sequences of enzymes involved in SA biosynthesis retrieved from *A. thaliana*, soybean and rice as queries. The table is split into the proteins part of the PAL pathway on the left side and the IC pathway on the right side. We used default pBLAST parameters and an E-value threshold of E ≤ 1e-6. For each SA biosynthesis protein, the orthologue with the lowest E-value is shown. Empty cells (indicated with ‘-‘) represent the absence of orthologues for the respective protein. For *T. virens*, the candidates selected for functional characterization are indicated in bold (TRIVIDRAFT_67832 and TRIVIDRAFT_47880)

### Optimization of a new KO approach

**Fig. 3:**
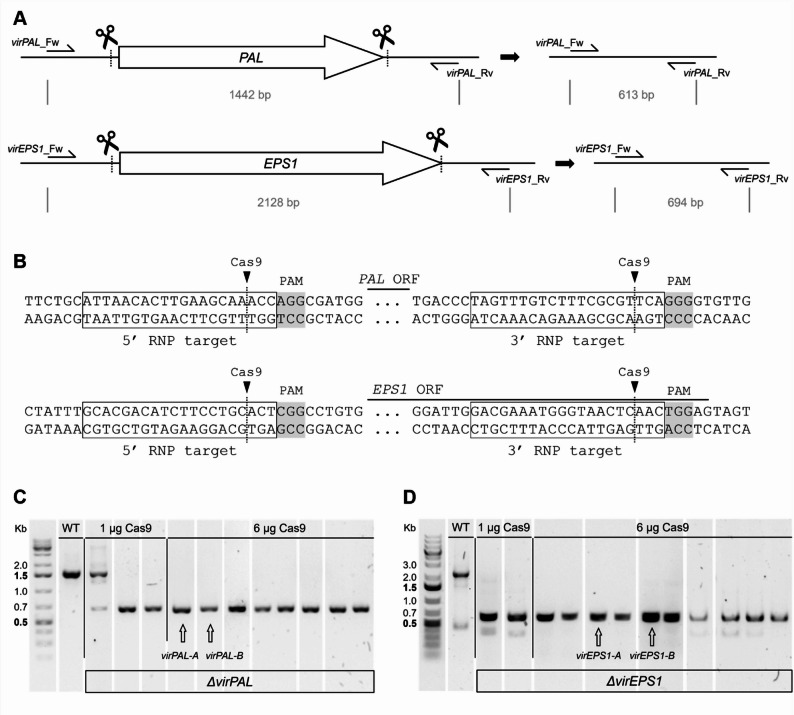
Genetic transformation for *virPAL* and *virEPS1* gene deletion in *T. virens*. **A** Experimental scheme indicating the CRISPR/Cas9 cut sites and the amplification fragment sizes for the *T. virens* WT and mutant genotypes. **B** Partial sequences up- and downstream of the *virPAL* and *virEPS1* open reading frames (ORF), showing the sequences targeted by the RNP complex (framed). The Cas9 cutting site is indicated with a triangle and the PAM sequence is highlighted in grey. **C**, **D** Summary of the genotyping of transformants emerging from the gene editing approach for deletion of *virPAL* (**B**) and *virEPS1* (**C**) using 1 µg and 6 µg Cas9. All positive mutants for each Cas9 quantity are shown in comparison to the WT. Mutants selected for further phenotyping are indicated with an arrow. The complete genotyping result is given in supplementary Figure S7

Deletion of *virPAL* and *virEPS1* in *T. virens* was achieved through application of an improved genetic transformation approach using CRISPR/Cas9 RNPs and a vector carrying a hygromycin B resistance gene (pTEL vector). CRISPR/Cas9 induces double-stranded breaks at both ends of the target gene, leading to gene deletion, while insertion of the pTEL vector provides resistance to hygromycin B, allowing transformant selection. Here, two Cas9 quantities were compared for their efficiency while the pTEL vector quantity was held constant. Although the lower amount of Cas9 (1 µg) resulted in more transformants for both target genes in both performed transformations, the higher Cas9 amount of 6 µg yielded more transformants that showed successful deletion of the target gene (Fig. 3). For the approach aiming at *virPAL* deletion, the use of 1 µg Cas9 yielded 32 transformants. Three of those were positive of which one heterokaryotic transformant contained both WT nuclei as well as nuclei with the *ΔvirPAL* genotype (9% positive, Fig. 3, B). The use of 6 µg Cas9 reduced the number of transformants to 21 but yielded eight positive homokaryotic transformants with a *ΔvirPAL* genotype (38% positive, Fig. 3, B). For *virEPS1* gene deletion, 25 transformants were obtained using 1 µg Cas9 with two positive homokaryotic transformants (8% positive, Fig. 3, C). The use of 6 µg Cas9 reduced the number of transformants to 19 but yielded 10 homokaryotic transformants with the *ΔvirEPS1* genotype (53% positive, Fig. 3, C). These data indicate an increased gene deletion frequency by using 6 µg Cas9. For both genes, two deletion mutants emerging from the transformation including 6 µg Cas9 were selected for further investigation (indicated in Fig. 3).

One of the advantages of the used transformation approach is the option to recycle the pTEL vector for repeated transformations using the same mutant. To test this mechanism in *T. virens*, we assessed the ability of the selected *ΔvirPAL* and *ΔvirEPS1* mutants to eliminate the pTEL vector. We observed that all four mutants (*ΔvirPAL-A*, *ΔvirPAL-B*, *ΔvirEPS1-A* and *ΔvirEPS1-B)* were able to eliminate the pTEL vector and required up to three cycles of cultivation on non-selective medium to do so. For the *ΔvirEPS1* mutants, one cycle of cultivation on PDA was sufficient while mutant *ΔvirPAL-A* required three cycles and *ΔvirPAL-B* two cycles.

### Mutant phenotyping

**Fig. 4:**
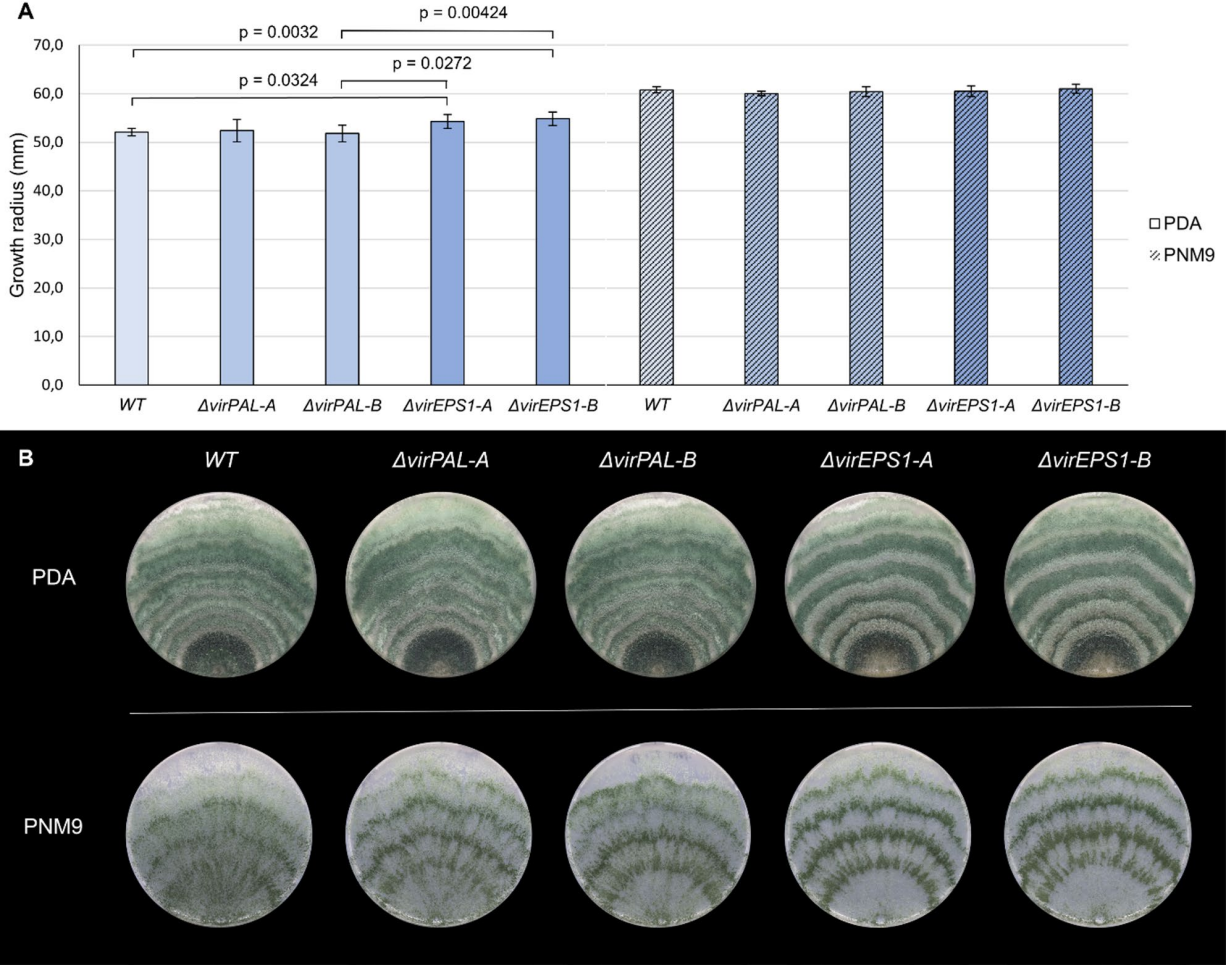
Phenotypical characterization of gene deletion mutants. Growth assay comparing the *ΔvirPAL* mutants (*ΔvirPAL-A* and *ΔvirPAL-B*) and *ΔvirEPS1* mutants (*ΔvirEPS1-A* and *ΔvirEPS1-B*) to *T. virens* WT on PDA and PNM9 solid medium. **A** The bar chart shows the average colony radius (n = three replicates) after five days of cultivation. Error bars represent the standard deviation. Statistically significant differences are indicated with brackets and p-values. **B** A representative colony for each strain is shown to assess the sporulation pattern after nine days of cultivation

Colony extension and sporulation of two independent *ΔvirPAL* and *ΔvirEPS1* mutants were compared to WT *T. virens* on PDA and PNM9 medium (Fig. 4). Deletion of *virPAL* did not have a significant effect on colony extension while deletion of *virEPS1* resulted in an increased colony radius on PDA but not on PNM9. The sporulation pattern of the *ΔvirEPS1* mutants differed slightly from that of the WT and *ΔvirPAL* mutants with more distinct lines of alternating darker and lighter pigmentation.

**Fig. 5:**
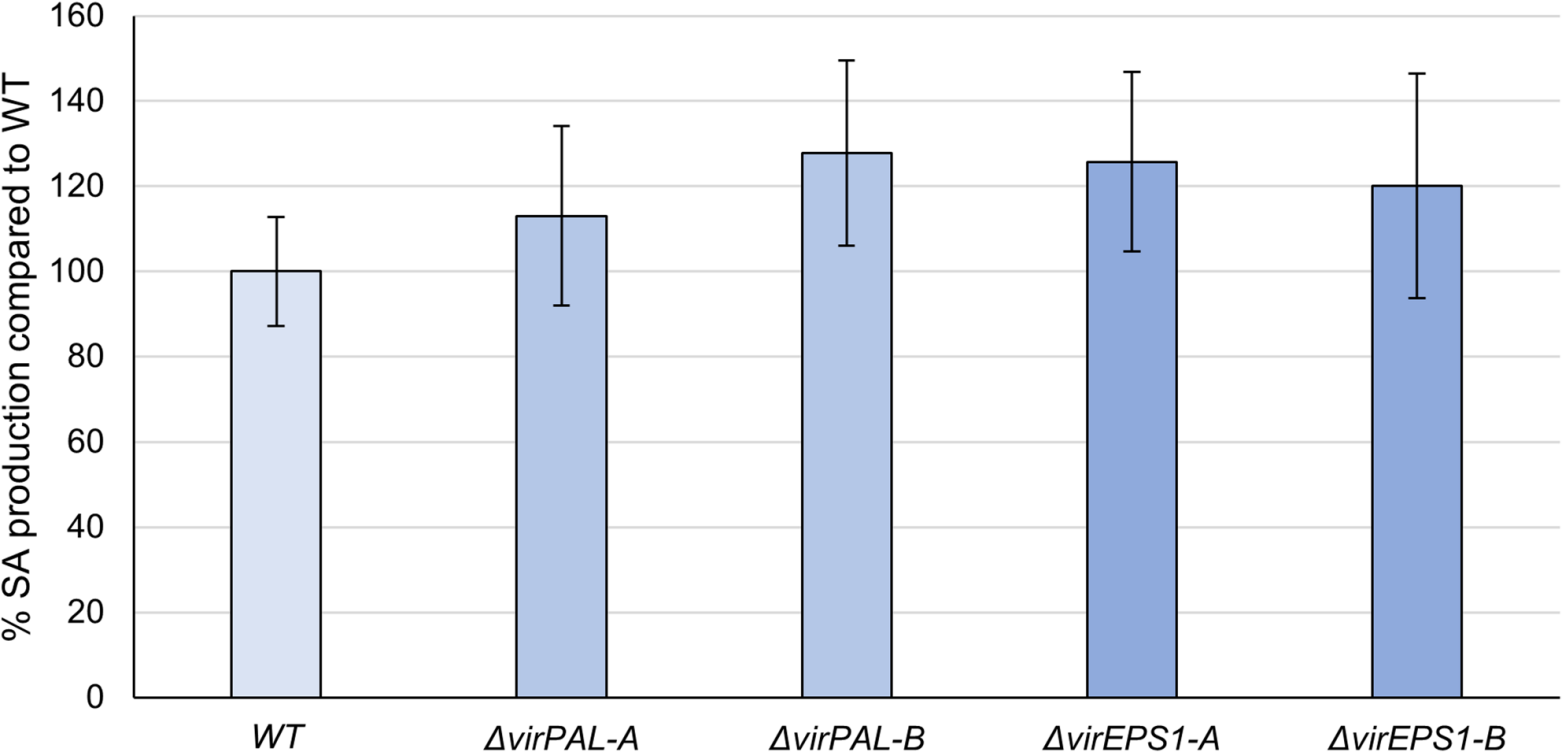
SA quantification for gene deletion mutants. Comparative assessment of SA biosynthesis by *T. virens* WT and *DvirPAL* and *ΔvirEPS1* mutants through UHPLC-MS/MS analysis. The amount of SA released into the culture medium by two independent *ΔvirPAL* mutants (*ΔvirPAL-A* and *ΔvirPAL-B*) and *ΔvirEPS1* mutants (*ΔvirEPS1-A* and *ΔvirEPS1-B*) is shown as a percentage relative to the SA released by *T. virens* WT. Error bars represent the standard deviation. No significant difference in extracellular SA were found between *T. virens* WT and *ΔvirPAL* or *ΔvirEPS1* mutants (*p* < 0.05)

The comparison of the amount of SA released by axenic cultures of *T. virens* WT and two independent *virPAL* (*ΔvirPAL-A* and *ΔvirPAL-B*) and *virEPS1* (*ΔvirEPS1-A* and *ΔvirEPS1-B*) gene deletion mutants grown on PNM9 revealed no significant differences (Fig. 5).

## Discussion

In this study, we investigated the influence of exogenous SA on the colony development of *Trichoderma* spp., quantified SA production and investigated the genetic basis of its biosynthesis. Significant species- and strain-dependent differences in responses to SA and in SA biosynthesis were found among the three *Trichoderma* species tested. Furthermore, an enhanced transformation approach was optimized for *T. virens* but provided no evidence that SA biosynthesis pathways are conserved between *T. virens* and plants.

The growth behaviour of the tested *Trichoderma* strains varied greatly in the presence of exogenous SA, with *T. virens* and *T. atroviride* P1 showing increased radial growth at low SA concentrations (25 µg mL^− 1^ to 100 µg mL^− 1^ for *T. atroviride* P1 and 50 µg mL^− 1^ for *T. virens*), whereas *T. atroviride* IMI 206040 exhibited decreased colony sizes and *T. asperellum* remained largely unaffected. The stimulation of colony extension may be induced by SA-triggered signalling pathways. Given the role of SA in plant immune responses, the observed growth increase could signify *Trichoderma* sensing stressed plants or potential host fungi and responding accordingly. Alternatively, these results might indicate the ability of some *Trichoderma* spp. to metabolize SA, as proposed for *Moniliophthora perniciosa* [62]. The SA-induced inhibition of *R. solani* growth supports the notion of SA’s antifungal properties, although the absence of such inhibition for *F. oxysporum* and the high SA concentrations used here suggest limited applicability of SA as a tool for *Trichoderma* to directly weaken host fungi.

The quantitative analysis of SA production revealed that all tested *Trichoderma* strains released extracellular SA with *T. virens* releasing the most. SA production had previously been reported in *T. harzianum*, *T. virens*, *T. longibrachiatum*, *T. parareesei*, *T. spirale* and *T. simmonsii*, but in none of the *Trichoderma* strains analysed here [16, 22]. Furthermore, we observed that VOCs from *A. thaliana* induced significant SA release in some *Trichoderma* spp., more so than direct physical contact. This observation could indicate that SA production by *Trichoderma* plays a role in initial plant interaction stages [20, 21, 27]. However, upon physical contact, *Trichoderma* might downregulate its endogenous SA production to facilitate symbiosis, in agreement with Morán-Diez et al. [27], who found that *Trichoderma* has to downregulate plant SA biosynthesis for optimal plant colonization. Alternatively, *A. thaliana* may have broken down the SA released by *Trichoderma.*

Notably, *T. atroviride* strains P1 and IMI 206040 differed significantly in their growth response to exogenous SA as well as in their SA release capacity. *T. atroviride* IMI 206040 showed inhibited growth in the presence of SA, whereas *T. atroviride* P1 demonstrated enhanced colony growth in the presence of up to 100 µg mL^− 1^ SA. Quantitative analysis of SA released by the different fungal strains showed significantly higher SA production by *T. atroviride* P1 compared to strain IMI 206040. We previously identified differences in light-mediated responses between these strains [63]. Altogether these findings underscore the need for careful strain selection in experimental designs, as even different strains and isolates of the same species may exhibit divergent physiological traits.

SA quantification was performed on solid PNM9 medium and included *A. thaliana*-*Trichoderma* co-cultivation. Traditionally, MS medium (0.2x or 1x) is used for co-cultivation of *Trichoderma* spp. with *A. thaliana* [64–67]. By combining modified PNM medium [46] and the glucose concentration from modified M9 medium [68] to create PNM9, we were able to create a medium that allowed similar or improved plant growth and improved fungal growth, compared to the traditional MS medium (data not shown). The addition of 0.5% glucose allows healthy fungal germling development and better replicates the natural soil environment where C-sources would be available [68]. In other studies, 0.6% to 0.75% sucrose were added to MS medium for co-cultivation experiments, likely to achieve the same result [69, 70]. Previous studies on phytohormone production in *Trichoderma* spp. quantified phytohormone levels upon fungal growth in liquid cultures [16, 22, 64, 70–72]. In this study, we quantified SA production by *Trichoderma* upon cultivation in solid culture, offering two key advantages over liquid cultures. First, the solid culture better replicates the natural soil environment of the fungus. Second, our approach allows more flexibility in experimental design. For example, our set-up allows co-cultivation of *Trichoderma* with a plant or host fungus. As a result, this is the first investigation into the effects of the presence of a plant on SA production by different *Trichoderma* species.

In contrast to the well-characterized pathways of SA biosynthesis in plants [28], the biosynthetic routes for SA production by fungi remain largely unknown. Our genetic and phylogenetic analyses investigated a potential conservation of SA biosynthesis pathways between plants and *Trichoderma* spp. The absence of a *PAL* orthologue in *T. asperellum*, despite its ability to produce SA, suggests that the PAL pathway is not involved. Interestingly, both *T. harzianum* and *T. asperellum* lack a *PAL* orthologue, despite their phylogenetic clustering with *PAL*-orthologue-containing *T. virens* and *T. atroviride*, respectively [73]. Similarly, the absence of orthologues of the plant genes *EDS5* and *PBS3*, essential for SA export and synthesis via the IC pathway in plants [28, 30], suggests that fungi might use alternative pathways or mechanisms for SA biosynthesis and transport. Nevertheless, to investigate this further, we chose *virPAL* and *virEPS1* as targets for gene deletion in *T. virens*, which in plants are part of the PAL and IC pathways for SA biosynthesis. Regarding the PAL pathway, the *PAL* gene was selected as there is a single *PAL* orthologue across *A. thaliana*, rice, and soybean genomes, while multiple orthologues for *AIM1*, encoding an enzyme that acts downstream, are encoded in the mentioned plants. Similarly, a single *EPS1* orthologue was identified across *A. thaliana*, rice, and soybean genomes and selected for gene deletion.

Using our optimized transformation approach, the selected target genes were successfully deleted in *T. virens*, the *Trichoderma* species with the highest SA production in our study. Previously, 27 µg of Cas9 were used for genome editing approaches in *T. atroviride* P1 [45]. However, Leisen et al. [43] showed that 6 µg Cas9 is sufficient for transformation of *B. cinerea*. We compared 1 µg and 6 µg of Cas9 and obtained a higher percentage of gene deletion mutants with 6 µg Cas9 compared to the lower amount of the endonuclease (38% compared to 9% for *ΔvirPAL* and 53% compared to 8% for *ΔvirEPS1)*. Furthermore, the pTEL vector used for transformant selection was eliminated in the mutants after a maximum of three rounds of cultivation on non-selective medium. Overall, this genetic manipulation approach offers several advantages over the traditional homologous recombination-based approach, including marker recycling, the removal of the need to design DNA fragments for HR, and elimination of potential confounding effects from genomically integrated resistance markers [45]. Given its novelty for application in *Trichoderma* species, optimization is crucial to improve efficiency and reduce costs. By applying this transformation approach to a new *Trichoderma* species and investigating optimal Cas9 quantities, we facilitate its future application.

Phenotypical characterization of the gene deletion mutants provided no evidence that a PAL or IC pathway is present or contributes to SA biosynthesis in *T. virens.* Neither the *ΔvirPAL* nor *ΔvirEPS1* mutants showed a significant change in SA production, suggesting either that the IC and PAL pathways are absent in *T. virens* or do not directly contribute to SA biosynthesis, or that redundancy exists between these pathways. To fully exclude the possibility of redundancy between a PAL and IC pathway in *T. virens*, a double KO mutant would need to be established.

Given these findings, a subsequent avenue of investigation could focus on putative orthologues of bacterial chorismate-derived pathways in *Trichoderma* species. In bacteria, SA is predominantly synthesized from chorismate via two well-characterized routes. The first involves a two-enzyme pathway, in which chorismate is converted to isochorismate by an isochorismate synthase (PchA-type), followed by cleavage of isochorismate to salicylic acid and pyruvate by an isochorismate-pyruvate lyase (PchB-type). This pathway is best described in several Gram-negative bacteria, where SA serves as a precursor for siderophore biosynthesis. The second route involves a single-enzyme salicylate synthase, typified by MbtI-like enzymes, which catalyse the direct conversion of chorismate to salicylic acid, without the release of a free isochorismate intermediate [29, 74, 75]. The absence of plant-like SA biosynthesis routes in *T. virens* raises the possibility that analogous chorismate-derived mechanisms may operate in *Trichoderma* species and potentially in fungi more broadly.

## Conclusions

The broader role of SA in symbiotic interactions remains contentious. The structural identity between plant and fungal SA has led to the hypothesis of a role of fungal SA in plant-fungal crosstalk [16, 20, 21]. Nevertheless, fungal SA could potentially activate SAR within plants and thereby hinder early symbiosis. Fungal SA might also play a role independent of plant interaction, potentially contributing to siderophore production for iron acquisition, as observed in bacteria [29]. In this context, investigating bacterial-inspired chorismate-derived SA biosynthesis pathways represents a potential avenue for future research in *Trichoderma*. Overall, our findings lay a foundation for further exploration of SA’s role in *Trichoderma* physiology, with potential implications for understanding fungal phytohormone production and for enhancing agricultural applications. Furthermore, by providing an optimized SA quantification and genomic manipulation approach, we facilitate future exploration into SA biosynthesis within *Trichoderma* species.

## Electronic Supplementary Material

Below is the link to the electronic supplementary material.

Supplementary Material 1.

Supplementary Material 2.

## Data Availability

The datasets used and/or analysed during the current study are available from the corresponding author on reasonable request.
