# Supplementary material for: Exploring salicylic acid biosynthesis in Trichoderma spp. using an enhanced transformation approach
Source: Fungal Biol Biotechnol. 2026 Feb 10;13:3. doi: 10.1186/s40694-026-00208-0 (PMC12930902; doi:10.1186/s40694-026-00208-0)
Supplement: Supplementary file 1 — Supplementary Material 1. [file 40694_2026_208_MOESM1_ESM.zip › Supplementary figures/Supplementary_Figures_Legends (2).docx]

FigureS1.png

Figure S1: Depiction of the cultivation set-ups used for SA quantification

Three cultivation setups were used for quantification of SA produced by Trichoderma spp. (A-D) Split-cultures were grown in a dish sandwich design to allow SA quantification of a Trichoderma strain (here T. atroviride IMI 206040) interacting with VOCs produced by A. thaliana without physical contact. The lid of a petri dish with a hole cutout was added in between to prevent the fungus from reaching the plant plate. (E) Co-culture in which the fungus (here T. atroviride P1) was grown with A. thaliana in the same plate. (F) Axenic culture which consists solely of one of the Trichoderma spp. (here T. asperellum). A 3D printed ‘cellophane fixing ring’ (inncellys GmbH, Mils, AT) was added to each plate to prevent the fungus from reaching the medium underneath the cellophane.

FigureS2.png

Figure S2: Phylogenetic analysis of the putative PAL and EPS1 orthologues across the genus *Trichoderma*

Putative or characterized PAL (A) or EPS1 (B) orthologues were identified using pBLAST and the respective identified orthologue sequence in *T. virens* (accession number XP_013953611.1 and XP_013953832.1/UKZ83807.1, respectively) as query. The maximum-likelihood phylogenetic trees derived show the evolutionary relationships of PAL and EPS1 within the genus. Bootstrap values are indicated as branch support only for the interval between 90 and 100. *Aspergillus tanneri* and *Beauveria bassiana* were used as outgroup for the PAL and EPS1 trees, respectively.

FigureS3.png

Figure S3: Cross-kingdom phylogenetic placement of *Trichoderma* PAL-like proteins

The putative PAL orthologue protein from *T. virens* was used to retrieve representative homologues from fungi, plants, and bacteria, and a maximum-likelihood tree was constructed to visualize the broader evolutionary context of the protein family. The tree highlights the fungal-specific clustering of PAL homologues and their divergence from related proteins in other taxa. Bootstraps values are provided only for longer branches due to readability issues.

FigureS4.png

Figure S4: Cross-kingdom phylogenetic placement of *Trichoderma* EPS1-like proteins

The putative EPS1 orthologue protein from *T. virens* was used to retrieve representative homologues from fungi, plants, and bacteria, and a maximum-likelihood tree was constructed to visualize the broader evolutionary context of the protein family. The tree highlights the fungal-specific clustering of EPS1 homologues and their divergence from related proteins in other taxa. Bootstraps values are provided only for longer branches due to readability issues.

FigureS5.png

Figure S5: Domain conservation analysis for PAL-like proteins.

The putative PAL orthologous proteins identified in the phylogenetic analysis (top) were used in combination with protein domain identification and analysis to investigate possible conservation of domains as well as active-site conservation across species. Blue marks indicate protein sequences that were hidden for readability purposes, adding no extra information to the already presented ones. Sequences that are highlighted in black correspond to domains identified by InterPro. The consensus sequence below the alignment served to identify the predicted active site of the enzymes. As each protein in the alignment has its own domain and active site prediction, as example we are reporting the information for *A. thaliana*, which generally provides more information and domains than any other annotated species for this specific protein. The complete prediction list, with GO terms from InterPro analysis is provided in Supplementary Table S4.

FigureS6.png

Figure S6: Domain conservation analysis for EPS1-like proteins.

The putative EPS1 orthologous proteins identified in the phylogenetic analysis (top) were used in combination with protein domain identification and analysis to investigate possible conservation of domains as well as active-site conservation across species. Blue marks indicate protein sequences that were hidden for readability purposes, adding no extra information to the already presented ones. Sequences that are highlighted in black/pink correspond to transferase domains identified by InterPro (pink when identified only in Pfam and Gene3; black when identified as well with PANTHER). The consensus sequence below the alignment served to identify the predicted active sites of the enzyme. As each protein in the alignment has its own domain and active site prediction, as example we are reporting the information for *A. thaliana*, for consistency. The complete prediction list, with GO terms from InterPro analysis is provided in Supplementary Table S4.

FigureS7.png

Figure S7: Genotyping of virPAL and virEPS1 gene deletion mutants

Genotyping of transformants emerging from the gene editing approach aiming at deletion of virPAL (A & B) and virEPS1 (C & D) using 1 µg and 6 µg Cas9. The results of the first round of genomic transformations are shown in A & C. The results of the second round are shown in B & D. An upper band on the electrophoresis gel indicates a WT genotype while a lower band indicates the absence of the GOI. Mutants selected for phenotyping are indicated with an arrow.
