# Supplementary figures and images for: Exploring salicylic acid biosynthesis in Trichoderma spp. using an enhanced transformation approach
Source: Fungal Biol Biotechnol. 2026 Feb 10;13:3. doi: 10.1186/s40694-026-00208-0 (PMC12930902; doi:10.1186/s40694-026-00208-0)

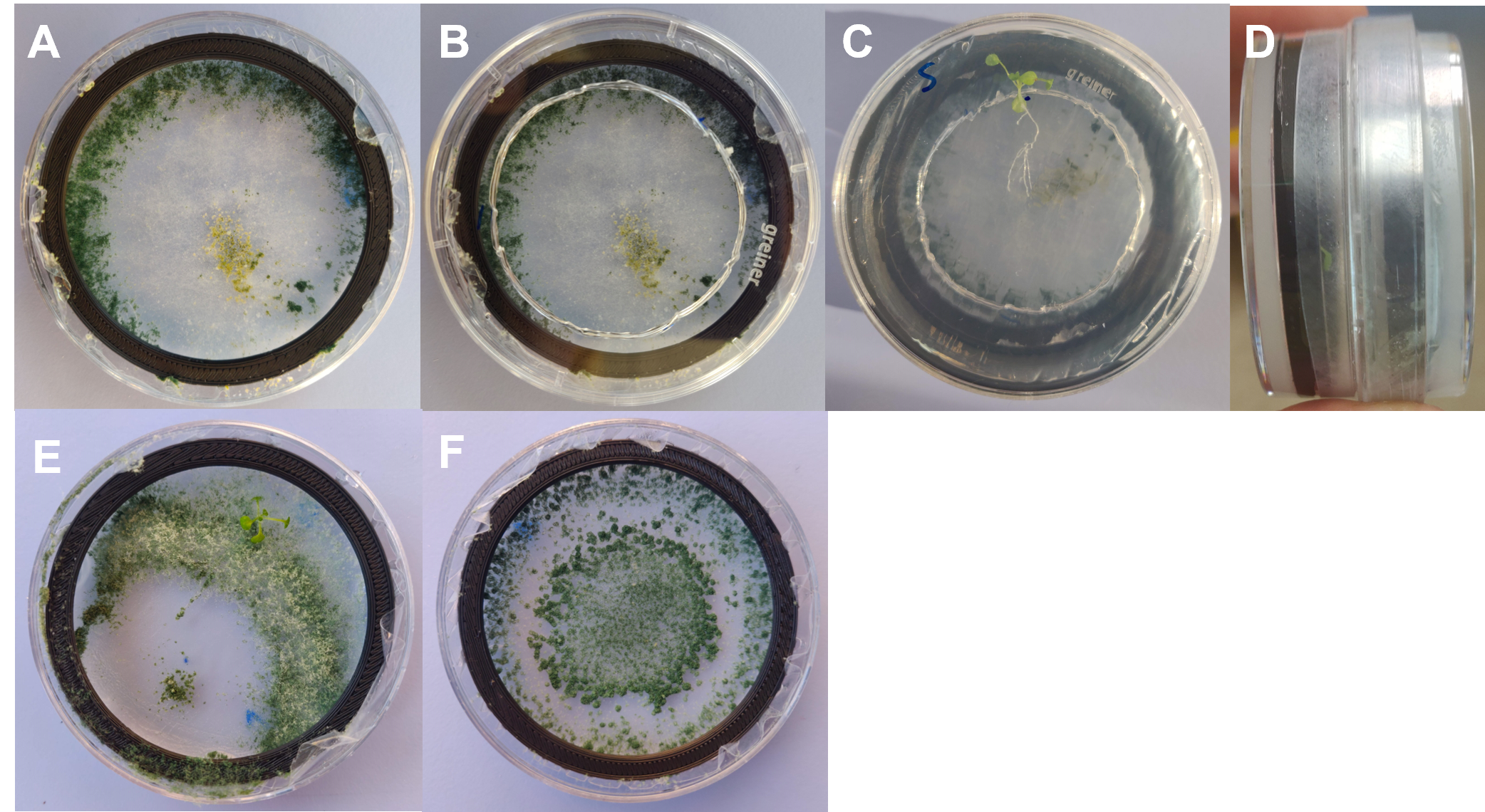

Supplement: Supplementary file 1 — Supplementary Material 1. [file 40694_2026_208_MOESM1_ESM.zip › Supplementary figures/FigureS1.png]

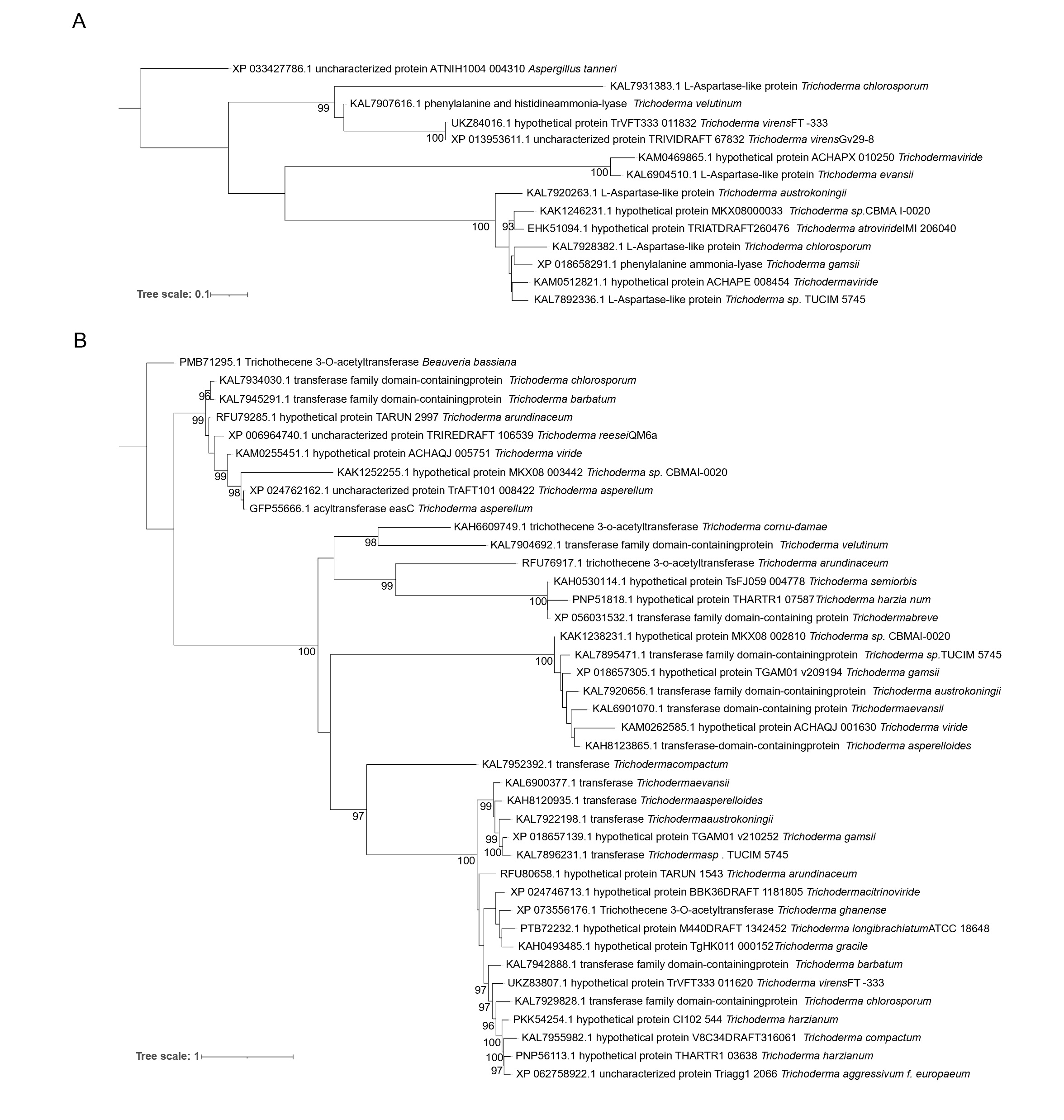

Supplement: Supplementary file 1 — Supplementary Material 1. [file 40694_2026_208_MOESM1_ESM.zip › Supplementary figures/FigureS2.png]

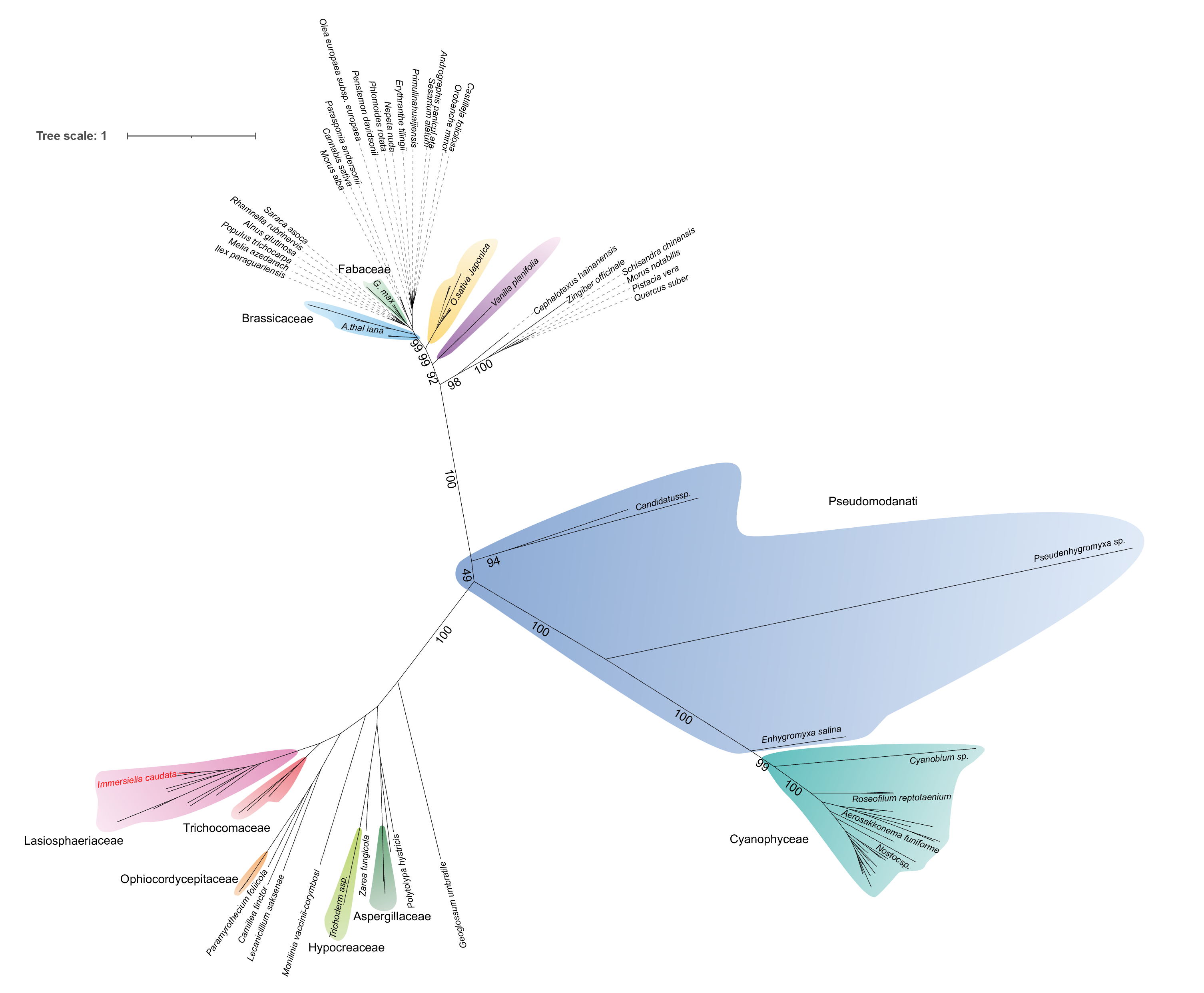

Supplement: Supplementary file 1 — Supplementary Material 1. [file 40694_2026_208_MOESM1_ESM.zip › Supplementary figures/FigureS3.png]

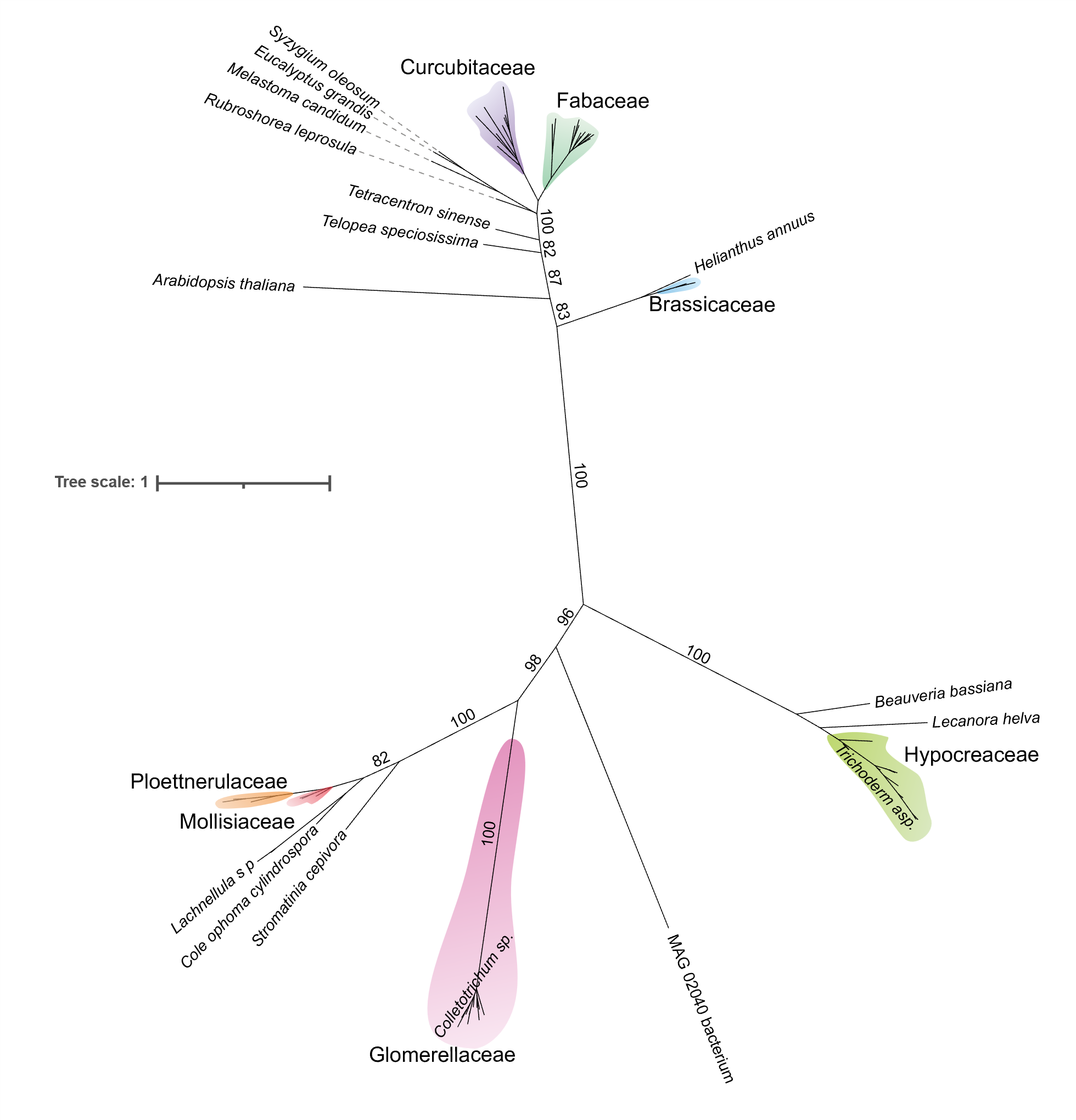

Supplement: Supplementary file 1 — Supplementary Material 1. [file 40694_2026_208_MOESM1_ESM.zip › Supplementary figures/FigureS4.png]

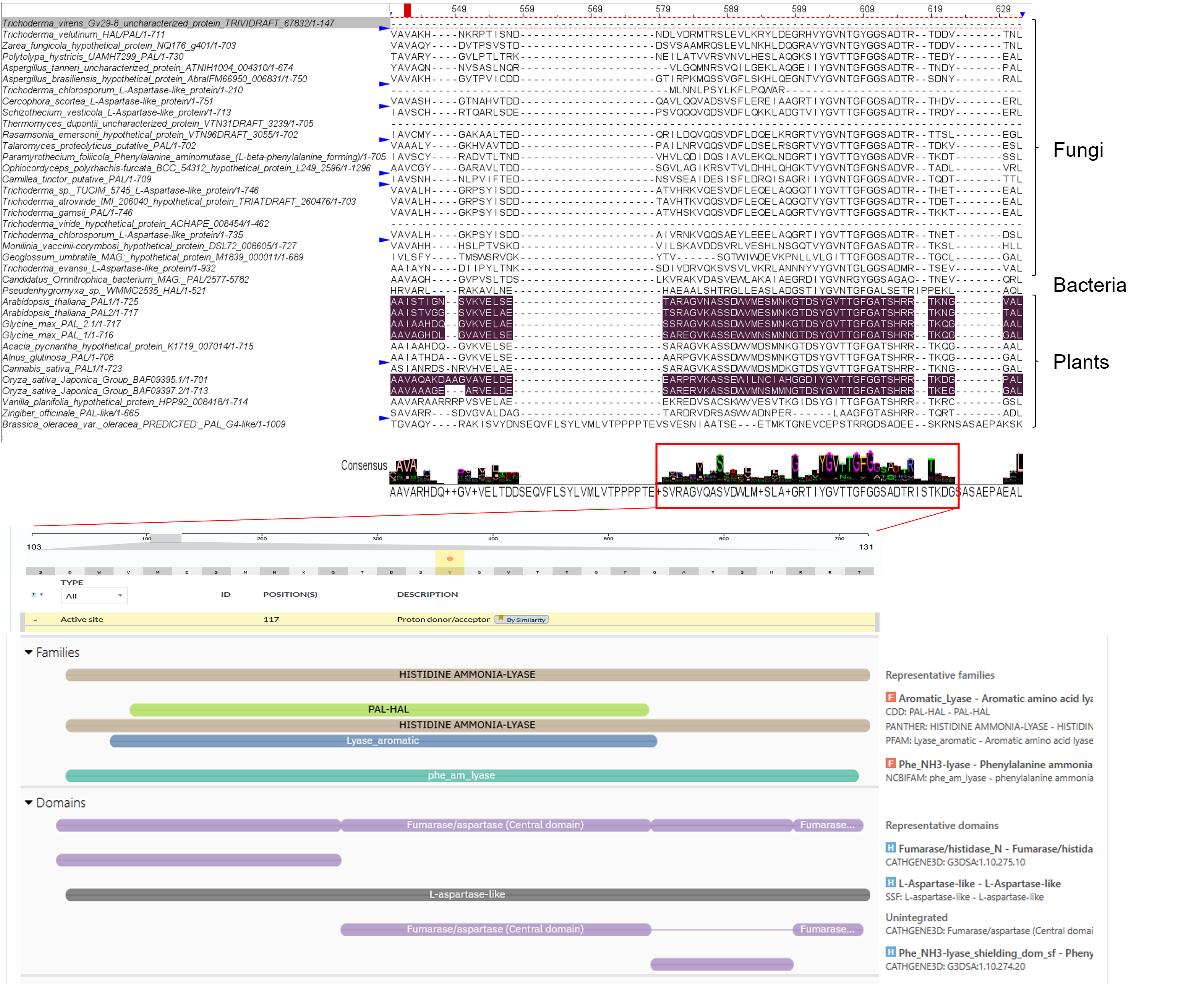

Supplement: Supplementary file 1 — Supplementary Material 1. [file 40694_2026_208_MOESM1_ESM.zip › Supplementary figures/FigureS5.png]

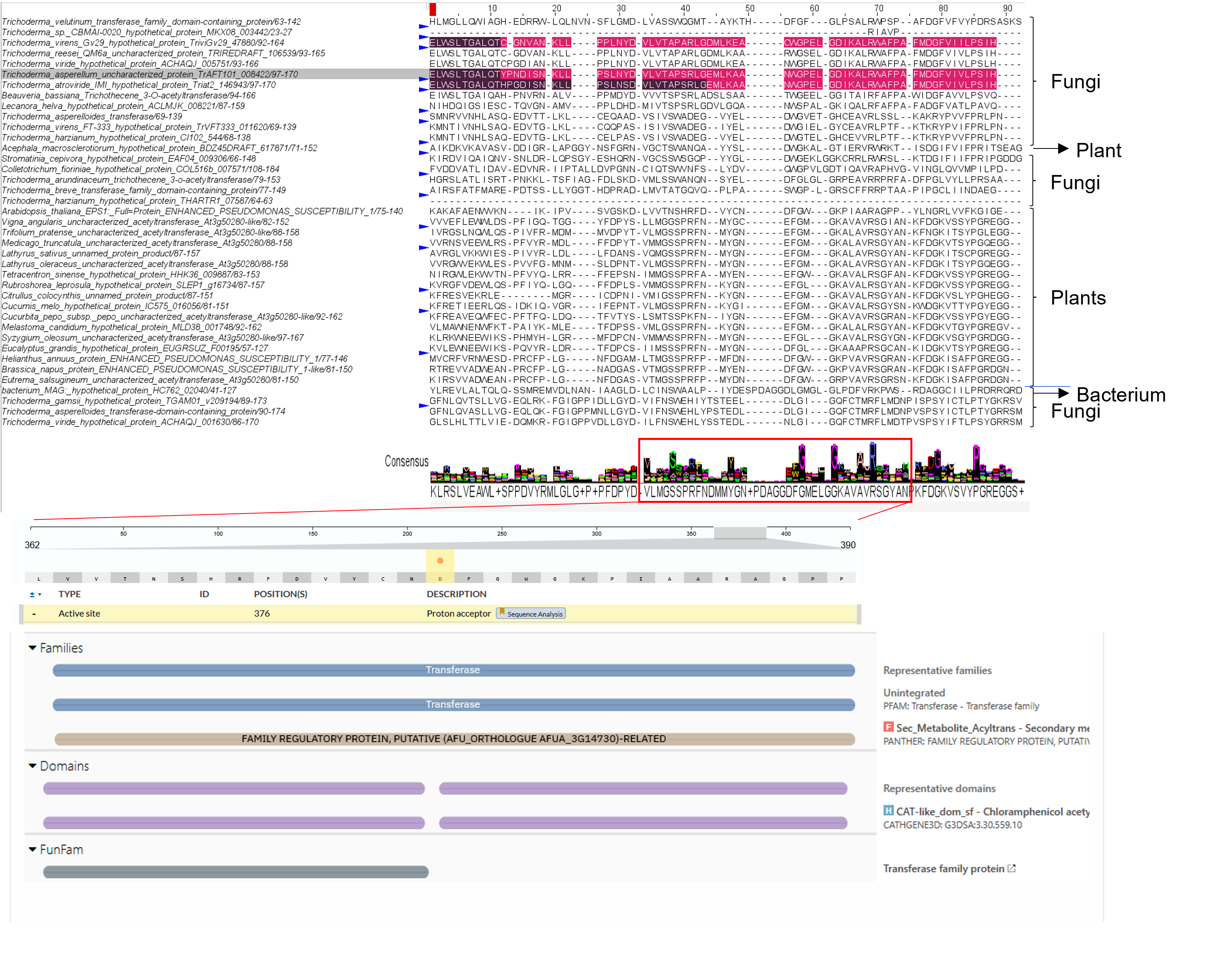

Supplement: Supplementary file 1 — Supplementary Material 1. [file 40694_2026_208_MOESM1_ESM.zip › Supplementary figures/FigureS6.png]

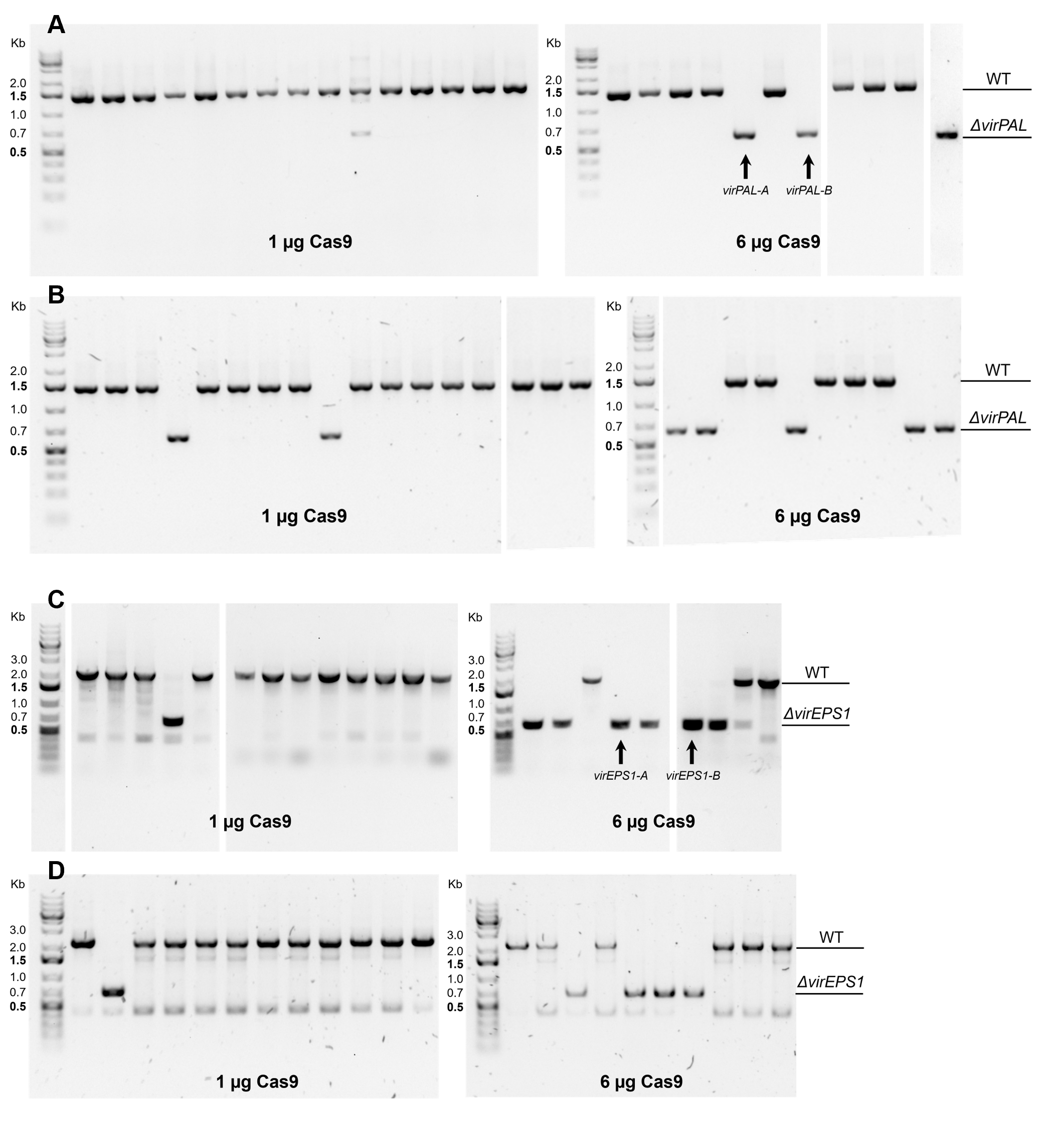

Supplement: Supplementary file 1 — Supplementary Material 1. [file 40694_2026_208_MOESM1_ESM.zip › Supplementary figures/FigureS7.png]
