# Supplementary material for: Exploring salicylic acid biosynthesis in Trichoderma spp. using an enhanced transformation approach
Source: Fungal Biol Biotechnol. 2026 Feb 10;13:3. doi: 10.1186/s40694-026-00208-0 (PMC12930902; doi:10.1186/s40694-026-00208-0)
Supplement: Supplementary file 2 — Supplementary Material 2. [file 40694_2026_208_MOESM2_ESM.zip › Supplementary tables/TableS1.docx]

Table S1: Complete summary of the pBLAST analysis results.

| ***Trichoderma* species** | **JGI catalog ID** | **Molecular function in Trichoderma** | **% Identity** | **% Coverage** | **E-value** | **Target annotation** | **Best hit** |
| --- | --- | --- | --- | --- | --- | --- | --- |
| *T. harzianum* | 510064 | 3-hydroxyacyl-CoA dehydrogenase | 33.33 | 38 | 1,00E-36 | Beta-oxidation fatty acids, conversion trans-cinnamic acid to benzoic acid (AIM1) | *A. thaliana* AT4G29010 |
|  | 215514 | Enoyl-CoA hydratase | 37.74 | 28 | 4,00E-28 |  |  |
|  | 107632 | Enoyl-CoA hydratase | 36.07 | 24 | 1,00E-26 |  |  |
|  | 507321 | Enoyl-CoA hydratase | 47.27 | 14 | 5,00E-21 |  |  |
|  | 10949 | Enoyl-CoA hydratase | 29.31 | 23 | 1,00E-18 |  |  |
|  | 91730 | Enoyl-CoA hydratase | 28.27 | 25 | 4,00E-16 |  |  |
|  | 512858 | Enoyl-CoA hydratase | 29.19 | 27 | 7,00E-15 |  |  |
|  | 101395 | Hydroxyacyl-CoA dehydrogenase/enoyl-CoA hydratase | 27.51 | 31 | 8,00E-15 |  |  |
|  | 112956 | 3-hydroxyacyl-CoA dehydrogenase | 29.41 | 20 | 6,00E-10 |  |  |
|  | 112315 | Isochorismate synthase / Anthranilate synthase | 31.16 | 29 | 2,00E-18 | Isochorismate synthase (ICS) | *A. thaliana* AT1G74710 (ICS1) |
|  | 112315 | Isochorismate synthase / Anthranilate synthase | 27.85 | 39 | 5,00E-17 | Isochorismate synthase (ICS) | *A. thaliana* AT1G18870 (ICS2) |
|  | 112315 | Isochorismate synthase / Anthranilate synthase | 25.29 | 40 | 1,00E-17 | Isochorismate synthase (ICS) | *O. sativa* Os09g19734 |
|  | 491874 | Porin/voltage-dependent anion-selective channel protein | 26.75 | 31 | 2,00E-10 |  |  |
|  | 112315 | Isochorismate synthase / Anthranilate synthase | 26.02 | 58 | 2,00E-25 | Isochorismate synthase (ICS) | *G. max* Glyma01g25690 |
|  | 112315 | Isochorismate synthase / Anthranilate synthase | 27.49 | 58 | 4,00E-26 | Isochorismate synthase (ICS) | *G. max* Glyma03g17420 |
|  | 505728 | Uncharacterized, contains CoA-dependant acyltransferase domain | 21.56 | 87 | 1,00E-11 | Conversion isochorismate-9-glutamate to salicylic acid (EPS1) | *A. thaliana* AT5G67160 |
| *T. asperellum* | 190397 | 3-hydroxyacyl-CoA dehydrogenase | 31.60 | 38 | 9,00E-35 | Beta-oxidation fatty acids, conversion trans-cinnamic acid to benzoic acid (AIM1) | *A. thaliana* AT4G29010 |
|  | 68668 | Putative enoyl-CoA hydratase | 34.42 | 29 | 1,00E-21 |  |  |
|  | 161768 | Putative enoyl-CoA hydratase | 36.11 | 22 | 3,00E-21 | Beta-oxidation fatty acids, conversion trans-cinnamic acid to benzoic acid (AIM1) | *A. thaliana* AT4G29010 |
|  | 146691 | Putative enoyl-CoA hydratase/isomerase | 29.44 | 25 | 5,00E-15 |  |  |
|  | 189343 | Isochorismate synthase / Anthranilate synthase | 31.16 | 29 | 8,00E-19 | Isochorismate synthase (ICS) | *A. thaliana* AT1G74710 (ICS1) |
|  | 189343 | Isochorismate synthase / Anthranilate synthase | 26.19 | 56 | 6,00E-18 | Isochorismate synthase (ICS) | *A. thaliana* AT1G18870 (ICS2) |
|  | 189343 | Isochorismate synthase / Anthranilate synthase | 28.94 | 27 | 1,00E-17 | Isochorismate synthase (ICS) | *O. sativa* Os09g19734 |
|  | 189343 | Isochorismate synthase / Anthranilate synthase | 27.19 | 58 | 9,00E-25 | Isochorismate synthase (ICS) | *G. max* Glyma01g25690 |
|  | 189343 | Isochorismate synthase / Anthranilate synthase | 26.90 | 58 | 9,00E-26 | Isochorismate synthase (ICS) | *G. max* Glyma03g17420 |
|  | 57229 | Uncharacterized, contains CoA-dependant acyltransferase domain | 21.04 | 87 | 1,00E-12 | Conversion isochorismate-9-glutamate to salicylic acid (EPS1) | *A. thaliana* AT5G67160 |
| *T. atroviride* | 260476 | Phenylalanine and histidine ammonia-lyase | 43.89 | 65 | 1,00E-118 | Phenylalanine ammonia-lyase (PAL) | *A. thaliana* AT2G37040 (PAL1) |
|  | 260476 | Phenylalanine and histidine ammonia-lyase | 43.17 | 66 | 6,00E-117 | Phenylalanine ammonia-lyase (PAL) | *A. thaliana* AT3G53260 (PAL2) |
|  | 260476 | Phenylalanine and histidine ammonia-lyase | 43.02 | 71 | 1,00E-116 | Phenylalanine ammonia-lyase (PAL) | *A. thaliana* AT5G04230 (PAL3) |
|  | 260476 | Phenylalanine and histidine ammonia-lyase | 43.13 | 70 | 4,00E-123 | Phenylalanine ammonia-lyase (PAL) | *A. thaliana* AT3G10340 (PAL4) |
|  | 260476 | Phenylalanine and histidine ammonia-lyase |  |  |  | Phenylalanine ammonia-lyase (PAL) | *O. sativa* Os02g41630 (PAL1) |
|  | 260476 | Phenylalanine and histidine ammonia-lyase |  |  |  | Phenylalanine ammonia-lyase (PAL) | *O. sativa* Os02g41650 (PAL2) |
|  | 260476 | Phenylalanine and histidine ammonia-lyase |  |  |  | Phenylalanine ammonia-lyase (PAL) | *O. sativa* Os02g41670 (PAL3) |
|  | 260476 | Phenylalanine and histidine ammonia-lyase |  |  |  | Phenylalanine ammonia-lyase (PAL) | *O. sativa* Os02g41680 (PAL4) |
|  | 260476 | Phenylalanine and histidine ammonia-lyase |  |  |  | Phenylalanine ammonia-lyase (PAL) | *O. sativa* Os04g43760 (PAL5) |
|  | 260476 | Phenylalanine and histidine ammonia-lyase | 44.58 | 66 | 5,00E-123 | Phenylalanine ammonia-lyase (PAL) | *O. sativa* Os04g43800 (PAL6) |
|  | 260476 | Phenylalanine and histidine ammonia-lyase |  |  |  | Phenylalanine ammonia-lyase (PAL) | *O. sativa* Os05g35290 (PAL7) |
|  | 260476 | Phenylalanine and histidine ammonia-lyase |  |  |  | Phenylalanine ammonia-lyase (PAL) | *O. sativa* Os11g48110 (PAL8) |
|  | 260476 | Phenylalanine and histidine ammonia-lyase |  |  |  | Phenylalanine ammonia-lyase (PAL) | *G. max* Glyma10g06600 (PAL) |
|  | 260476 | Phenylalanine and histidine ammonia-lyase | 42.86 | 65 | 1,00E-120 | Phenylalanine ammonia-lyase (PAL) | *G. max* Glyma19g36620 (PAL) |
|  | 260476 | Phenylalanine and histidine ammonia-lyase |  |  |  | Phenylalanine ammonia-lyase (PAL) | *G. max* Glyma03g33880 (PAL) |
|  | 260476 | Phenylalanine and histidine ammonia-lyase |  |  |  | Phenylalanine ammonia-lyase (PAL) | *G. max* Glyma13g20800 (PAL) |
|  | 260476 | Phenylalanine and histidine ammonia-lyase |  |  |  | Phenylalanine ammonia-lyase (PAL) | *G. max* Glyma02g47940 (PAL) |
|  | 267956 | 3-hydroxyacyl-CoA dehydrogenase | 31.60 | 38 | 3,00E-35 | Beta-oxidation fatty acids, conversion trans-cinnamic acid to benzoic acid (AIM1) | *A. thaliana* AT4G29010 |
|  | 301812 | Putative enoyl-CoA hydratase | 36.07 | 24 | 6,00E-26 |  |  |
|  | 35736 | hypothetical protein similar to enoyl-CoA hydratase | 40.00 | 16 | 4,00E-21 |  |  |
|  | 195394 | Putative enoyl-CoA hydratase/isomerase | 31.47 | 25 | 1,00E-15 |  |  |
|  | 199208 | Hydroxyacyl-CoA dehydrogenase/enoyl-CoA hydratase | 25.64 | 29 | 1,00E-13 |  |  |
|  | 297768 | Isochorismate synthase / Anthranilate synthase | 30.65 | 29 | 9,00E-19 | Isochorismate synthase (ICS) | *A. thaliana* AT1G74710 (ICS1) |
|  | 297768 | Isochorismate synthase / Anthranilate synthase | 26.19 | 56 | 2,00E-17 | Isochorismate synthase (ICS) | *A. thaliana* AT1G18870 (ICS2) |
|  | 297768 | Isochorismate synthase / Anthranilate synthase | 28.94 | 27 | 1,00E-17 | Isochorismate synthase (ICS) | *O. sativa* Os09g19734 (ICS) |
|  | 257201 | Eukaryotic porin | 26.94 | 31 | 1,00E-10 |  |  |
|  | 297768 | Isochorismate synthase / Anthranilate synthase | 27.27 | 58 | 3,00E-25 | Isochorismate synthase (ICS) | *G. max* Glyma01g25690 (ICS) |
|  | 297768 | Isochorismate synthase / Anthranilate synthase | 27.27 | 58 | 5,00E-27 | Isochorismate synthase (ICS) | *G. max* Glyma03g17420 (ICS) |
|  | 146943 | Uncharacterized, contains CoA-dependant acyltransferase domain | 22.12 | 88 | 7,00E-11 | Conversion isochorismate-9-glutamate to salicylic acid (EPS1) | *A. thaliana* AT5G67160 |
| *T. virens* | 67832 | Phenylalanine and histidine ammonia-lyase | 33.53 | 23 | 2,00E-16 | Phenylalanine ammonia-lyase (PAL) | *A. thaliana* AT2G37040 (PAL1) |
|  | 67832 | Phenylalanine and histidine ammonia-lyase | 32.35 | 23 | 6,00E-15 | Phenylalanine ammonia-lyase (PAL) | *A. thaliana* AT3G53260 (PAL2) |
|  | 67832 | Phenylalanine and histidine ammonia-lyase | 34.10 | 24 | 8,00E-15 | Phenylalanine ammonia-lyase (PAL) | *A. thaliana* AT5G04230 (PAL3) |
|  | 67832 | Phenylalanine and histidine ammonia-lyase | 34.48 | 23 | 3,00E-17 | Phenylalanine ammonia-lyase (PAL) | *A. thaliana* AT3G10340 (PAL4) |
|  | 67832 | Phenylalanine and histidine ammonia-lyase |  |  |  | Phenylalanine ammonia-lyase (PAL) | *O. sativa* Os02g41630 (PAL1) |
|  | 67832 | Phenylalanine and histidine ammonia-lyase |  |  |  | Phenylalanine ammonia-lyase (PAL) | *O. sativa* Os02g41650 (PAL2) |
|  | 67832 | Phenylalanine and histidine ammonia-lyase |  |  |  | Phenylalanine ammonia-lyase (PAL) | *O. sativa* Os02g41670 (PAL3) |
|  | 67832 | Phenylalanine and histidine ammonia-lyase |  |  |  | Phenylalanine ammonia-lyase (PAL) | *O. sativa* Os02g41680 (PAL4) |
|  | 67832 | Phenylalanine and histidine ammonia-lyase |  |  |  | Phenylalanine ammonia-lyase (PAL) | *O. sativa* Os04g43760 (PAL5) |
|  | 67832 | Phenylalanine and histidine ammonia-lyase | 31.55 | 23 | 4,00E-14 | Phenylalanine ammonia-lyase (PAL) | *O. sativa* Os04g43800 (PAL6) |
|  | 67832 | Phenylalanine and histidine ammonia-lyase |  |  |  | Phenylalanine ammonia-lyase (PAL) | *O. sativa* Os05g35290 (PAL7) |
|  | 67832 | Phenylalanine and histidine ammonia-lyase |  |  |  | Phenylalanine ammonia-lyase (PAL) | *O. sativa* Os11g48110 (PAL8) |
|  | 67832 | Phenylalanine and histidine ammonia-lyase |  |  |  | Phenylalanine ammonia-lyase (PAL) | *G. max* Glyma10g06600 (PAL) |
|  | 67832 | Phenylalanine and histidine ammonia-lyase |  |  |  | Phenylalanine ammonia-lyase (PAL) | *G. max* Glyma19g36620 (PAL) |
|  | 67832 | Phenylalanine and histidine ammonia-lyase | 33.33 | 23 | 1,00E-15 | Phenylalanine ammonia-lyase (PAL) | *G. max* Glyma03g33880 (PAL) |
|  | 67832 | Phenylalanine and histidine ammonia-lyase |  |  |  | Phenylalanine ammonia-lyase (PAL) | *G. max* Glyma13g20800 (PAL) |
|  | 67832 | Phenylalanine and histidine ammonia-lyase |  |  |  | Phenylalanine ammonia-lyase (PAL) | *G. max* Glyma02g47940 (PAL) |
|  | 211402 | 3-hydroxyacyl-CoA dehydrogenase | 31.94 | 38 | 1,00E-34 | Beta-oxidation fatty acids, conversion trans-cinnamic acid to benzoic acid (AIM1) | *A. thaliana* AT4G29010 |
|  | 50461 | Enoyl-CoA hydratase | 36.79 | 28 | 2,00E-27 |  |  |
|  | 87842 | Enoyl-CoA hydratase | 36.61 | 24 | 6,00E-27 |  |  |
|  | 178934 | Enoyl-CoA hydratase | 42.42 | 16 | 2,00E-21 |  |  |
|  | 51758 | Enoyl-CoA hydratase | 27.31 | 30 | 2,00E-18 |  |  |
|  | 68727 | Enoyl-CoA hydratase | 29.69 | 26 | 1,00E-16 |  |  |
|  | 216321 | Enoyl-CoA hydratase | 27.75 | 25 | 8,00E-16 |  |  |
|  | 59655 | 3-hydroxyacyl-CoA dehydrogenase | 29.00 | 26 | 1,00E-10 |  |  |
|  | 46955 | 3-hydroxyacyl-CoA dehydrogenase | 25.91 | 32 | 6,00E-10 |  |  |
|  | 84460 | Isochorismate synthase / Anthranilate synthase | 30.15 | 29 | 1,00E-17 | Isochorismate synthase (ICS) | *A. thaliana* AT1G74710 (ICS1) |
|  | 84460 | Isochorismate synthase / Anthranilate synthase | 27.43 | 39 | 1,00E-16 | Isochorismate synthase (ICS) | *A. thaliana* AT1G18870 (ICS2) |
|  | 84460 | Isochorismate synthase / Anthranilate synthase | 28.51 | 27 | 3,00E-17 | Isochorismate synthase (ICS) | *O. sativa* Os09g19734 (ICS) |
|  | 111054 | Porin/voltage-dependent anion-selective channel protein | 26.57 | 31 | 2,00E-10 |  |  |
|  | 84460 | Isochorismate synthase / Anthranilate synthase | 26.90 | 58 | 8,00E-25 | Isochorismate synthase (ICS) | *G. max* Glyma01g25690 (ICS) |
|  | 84460 | Isochorismate synthase / Anthranilate synthase | 26.61 | 58 | 8,00E-26 | Isochorismate synthase (ICS) | *G. max* Glyma03g17420 (ICS) |
|  | 47880 | Uncharacterized, contains CoA-dependant acyltransferase domain | 21.13 | 87 | 2,00E-12 | Conversion isochorismate-9-glutamate to salicylic acid (EPS1) | *A. thaliana* AT5G67160 |

pBLAST analysis was performed in T. harzianum *CBS 226.95*, T. asperellum *CBS 433.97*, T. atroviride *IMI 206040* and T. virens *Gv29-8* with the protein sequences of enzymes involved in SA biosynthesis retrieved from *A. thaliana*, soybean and rice as queries. Default pBLAST parameters and an E-value threshold of E ≤ 1e-6 were used. For PAL of rice and soybean, the percentage identity, coverage and E- value is only given for the main PAL protein (Os04g43800 for rice and Glyma03g33880 for soybean)(Lefevere et al., 2020; Shine et al., 2016). No orthologs were found for *A. thaliana* EDS5 (AT4G39030) and PBS3 (AT5G13320).
