# Supplementary material for: Exploring salicylic acid biosynthesis in Trichoderma spp. using an enhanced transformation approach
Source: Fungal Biol Biotechnol. 2026 Feb 10;13:3. doi: 10.1186/s40694-026-00208-0 (PMC12930902; doi:10.1186/s40694-026-00208-0)
Supplement: Supplementary file 2 — Supplementary Material 2. [file 40694_2026_208_MOESM2_ESM.zip › Supplementary tables/TableS2.docx]

Table S2: CRISPR RNA and primer list

| **CRISPR-Cas9 crRNAs** | **Sequence (5’ to 3’)** |
| --- | --- |
| Upstream *virPAL* | ATTAACACTTGAAGCAAACCGTTTTAGAGCTATGCT |
| Downstream *virPAL* | TAGTTTGTCTTTCGCGTTCAGTTTTAGAGCTATGCT |
| Upstream *virEPS1* | GCACGACATCTTCCTGCACTGTTTTAGAGCTATGCT |
| Downstream *virEPS1* | GACGAAATGGGTAACTCAACGTTTTAGAGCTATGCT |
| **Primers** | **Sequence (5’ to 3’)** |
| *virPAL*_Fw | AGCCCACTGAACAATCCGTT |
| *virPAL*_Rv | CAGTAATGAGGGCCAGCGAA |
| *virEPS1*_Fw | AATAATCTCCTTGGCCGCCC |
| *virEPS1* _Rv | CCTCGTACTGACAGCAGGTG |

CRISPR RNAs used for targeting the GOIs to produce gene deletion mutants and primers used for genotyping of the obtained *T. virens* transformants.
