# Supplementary material for: Exploring salicylic acid biosynthesis in Trichoderma spp. using an enhanced transformation approach
Source: Fungal Biol Biotechnol. 2026 Feb 10;13:3. doi: 10.1186/s40694-026-00208-0 (PMC12930902; doi:10.1186/s40694-026-00208-0)
Supplement: Supplementary file 2 — Supplementary Material 2. [file 40694_2026_208_MOESM2_ESM.zip › Supplementary tables/TableS3.docx]

Table S3: detailed results statistical tests

| **Comparison** | **Statistical test** | **p-value** |
| --- | --- | --- |
| Figure 1, *T. atroviride* IMI206040, 0µg/mL – 25µg/mL | Welch’s ANOVA | 0.039 |
| Figure 1, *T. atroviride* IMI206040, 0µg/mL – 50µg/mL | Welch’s ANOVA | 0.003 |
| Figure 1, *T. atroviride* IMI206040, 25µg/mL – 50µg/mL | Welch’s ANOVA | 0.0472 |
| Figure 1, *T. atroviride* IMI206040, 25µg/mL – 100µg/mL | Welch’s ANOVA | 0.023 |
| Figure 1, *T. atroviride* IMI206040, 50µg/mL – 100µg/mL | Welch’s ANOVA | 0.017 |
| Figure 1, *T. atroviride* P1, 0µg/mL – 25µg/mL | Kruskal-Wallis test | 0.00077 |
| Figure 1, *T. atroviride* P1, 0µg/mL – 50µg/mL | Kruskal-Wallis test | 0.00077 |
| Figure 1, *T. atroviride* P1, 0µg/mL – 100µg/mL | Kruskal-Wallis test | 0.00077 |
| Figure 1, *T. asperellum*, 0µg/mL – 100µg/mL | One-way ANOVA | 1.19e-13 |
| Figure 1, *T. asperellum*, 25µg/mL – 100µg/mL | One-way ANOVA | 1.05e-13 |
| Figure 1, *T. asperellum*, 50µg/mL – 100µg/mL | One-way ANOVA | 1.09e-13 |
| Figure 1, *T. virens*, 0µg/mL – 50µg/mL | Welch’s ANOVA | 2e-7 |
| Figure 1, *T. virens*, 25µg/mL – 50µg/mL | Welch’s ANOVA | 2.16e-5 |
| Figure 1, *T. virens*, 50µg/mL – 100µg/mL | Welch’s ANOVA | 2e-7 |
| Figure 1, *F. oxysporum*, no significant differences | Kruskal-wallis test |  |
| Figure 1, *R. solani*, 0µg/mL – 50µg/mL | One-way ANOVA | 4.36e-4 |
| Figure 1, *R. solani*, 0µg/mL – 100µg/mL | One-way ANOVA | 1.17e-13 |
| Figure 1, *R. solani*, 25µg/mL – 50µg/mL | One-way ANOVA | 1.32e-6 |
| Figure 1, *R. solani*, 25µg/mL – 100µg/mL | One-way ANOVA | 1.01e-13 |
| Figure 1, *R. solani*, 50µg/mL – 100µg/mL | One-way ANOVA | 3.53e-10 |
| Figure 2, A, IMI – IMI+VOCs(A) | Kruskal-Wallis test | 0.017 |
| Figure 2, C, ASP – ASP+VOCs(A) | Kruskal-Wallis test | 0.017 |
| Figure 2, E, IMI – P1 | Kruskal-Wallis test | 0.017 |
| Figure 2, E, IMI – ASP | Kruskal-Wallis test | 0.023 |
| Figure 2, E, IMI – VIR | Kruskal-Wallis test | 0.017 |
| Figure 2, E, P1 – VIR | Kruskal-Wallis test | 0.017 |
| Figure 2, E, ASP – VIR | Kruskal-Wallis test | 0.017 |
| Figure 4, A, WT – *ΔvirEPS1-A* | One-way ANOVA | 0.0324 |
| Figure 4, A, WT – *ΔvirEPS1-B* | One-way ANOVA | 0.0032 |
| Figure 4, A, *ΔvirPAL-B* – *ΔvirEPS1-A* | One-way ANOVA | 0.0272 |
| Figure 4, A, *ΔvirPAL-B* – *ΔvirEPS1-B* | One-way ANOVA | 0.00424 |
| Figure 5, not significant differencs | Kruskal-Wallis test |  |

Detailed summary of applied statistical tests and resulting p-values.
